# A scoping review on adult patients with de novo glomerular diseases following COVID-19 infection or vaccine

**DOI:** 10.1007/s11255-024-04189-0

**Published:** 2024-09-03

**Authors:** Liam Qi, Aman Deep, Jordan Fox, Mark Yii, Muhammad Rahman, Mar Myint, Htoo Myat, Zaw Thet

**Affiliations:** 1 Department of Nephrology, Central Queensland Hospital and Health Service, Rockhampton, QLD Australia; 2 Department of Medicine, Prince Charles Hospital, Metro North Hospital and Health Service, Chermside, QLD Australia; 3 Department of Medicine, Flinders Medical Centre, South Adelaide Local Health Network, Bedford Park, SA Australia; 4 Department of Medicine, Canberra Hospital, Canberra Hospital and Health Service, Garran, ACT Australia; 5 K & K Kidney Health, Rockhampton, QLD Australia; 6 The University of Queensland Rural Clinical School, Rockhampton, QLD Australia; 7 School of Medicine and Dentistry, Menzies Health Institute Queensland, Griffith University, Gold Coast, QLD Australia; 8 The University of Queensland, Faculty of Medicine, Brisbane, QLD Australia

**Keywords:** COVID-19 infection, COVID-19 vaccination, Glomerulonephritis, Glomerulopathy, Glomerular disease

## Abstract

**Background:**

There are increasing reports of glomerular disease (GD) following COVID-19 infection and vaccination. Current evidence on the possible link between COVID-19 infection or vaccination and GD is conflicting.

**Objective:**

The present study undertakes a scoping review of research to describe the relationship between COVID-19 infection and vaccination with GD and the common management strategies and overall outcomes of the disease to identify knowledge gaps and guide further research.

**Eligibility criteria:**

All original research studies published in English until 5th September 2022 were considered for inclusion in the review. Exclusion criteria were animal studies, autopsy studies, and data involving patients who were paediatric patients (< 16 years), were transplant recipients, had a recurrence of glomerular disease, had concomitant cancer or non-COVID-19 infection which may cause glomerular disease, or did not receive a renal biopsy.

**Sources of evidence:**

The five electronic databases searched were MEDLINE, PubMed, Scopus, EMBASE, and Cochrane.

**Methods:**

Two separate search strings related to COVID-19, and glomerular disease were combined using the Boolean operator ‘AND’. Filters were used to limit publications to original research studies published in English. Search results from each database were imported into Covidence software (www.covidence.org) and used for de-duplication, article screening, and data extraction. Descriptive analyses were used to summarise demographics, diagnoses, and treatment outcomes.

**Results:**

After removing duplicates, 6853 titles and abstracts were screened. Of the 188 studies included, 106 studies described 341 patients with GD following COVID-19 infection and 82 described 146 patients with GD following a COVID-19 vaccination. IgA nephropathy was the most common GD pathology reported following COVID-19 vaccination with GD most common following mRNA vaccines. Collapsing focal segmental glomerulosclerosis was the most common GD following COVID-19 infection. Immunosuppressive treatment of GD was more common in the vaccine cohort than in the infection cohort.

**Conclusion:**

Despite the significant number of COVID-19 infections and vaccinations around the world, our understanding of GD associated with COVID-19 infection and vaccination remains poor, and more research is needed to understand the possible relationship better.

**Supplementary Information:**

The online version contains supplementary material available at 10.1007/s11255-024-04189-0.

## Background

Coronavirus disease 2019 (COVID-19) caused by Severe Acute Respiratory Syndrome-Coronovirus-2 (SARS-CoV-2) has resulted in the largest pandemic of the last century [1]. To date, there have been 697,096,497 confirmed cases of COVID-19 leading to 6,932,514 deaths [2]. Over 13.5 billion doses of the COVID-19 vaccine have been administered globally, with 70.6% of the world's population receiving at least one dose [3]. Renal pathologies have been recorded post-administration of these vaccinations with similar reports in patients with COVID-19 infection [4, 5]. In patients hospitalised with COVID-19 infection, the prevalence of acute kidney injury (AKI) was 28%, which increased to 46% of patients admitted to the intensive care unit, and the prevalence of patients requiring kidney replacement therapy was 9% [6].

Glomerular disease (GD) is the most common cause of chronic kidney disease (CKD) leading to end-stage kidney disease (ESKD) requiring renal replacement therapy after diabetes [7–9]. Development of de novo and recurrent GD following COVID-19 infection or vaccination are increasingly reported in the literature with various pathogenic mechanisms proposed [10]. Many studies have attempted to describe the possible association between COVID-19 infection and vaccination with GD, resulting in conflicting reports [10–13]. Significant morbidity has been described with between 9–70% of these patients requiring acute dialysis and up to 6–30% of patients needing to remain on dialysis long-term [10–13].

This scoping review aims to describe the characteristics of patients who develop de novo GD following COVID-19 infection or vaccination and the overall treatment decisions and outcomes to identify knowledge gaps and future research directions.

## Methods

### Study design

This scoping review was conducted following the JBI Manual for Evidence Synthesis guidelines and the Preferred Reporting items for systematic Reviews and Meta-Analyses Extension for Scoping Reviews (PRISMA-ScR) [14, 15]. All original research studies published in English until 5th September 2022 were considered for inclusion in the review. Exclusion criteria were animal studies, autopsy studies, and data involving paediatric patients (< 16 years), transplant recipients, patients with GD recurrence, patients with concomitant cancer or non-COVID-19 infection which may cause GD or patients who did not receive a renal biopsy. The abstract was also subsequently excluded if a conference abstract and case report presented the same patient.

Two experienced researchers (ZT, JF) drafted the search categories and further refined them through team discussion. A systematic search of five electronic databases (MEDLINE, PubMed, Scopus, EMBASE, and Cochrane) was conducted using two separate search strings related to COVID-19 (COVID, coronavirus, SARS-CoV-2) and glomerular disease (glomerular disease, glomerulopathy, glomerulonephritis, glomerulosclerosis, proteinuria, haematuria, acute kidney injury, AKI) were combined using the Boolean operator ‘AND’. Filters were used to limit publications to original research studies published in English. The complete search strategies for each database are presented in Supplementary Table 1.

### Data collection

Search results from each database were imported into Covidence software (www.covidence.org) and used for de-duplication, article screening, and data extraction. The title and abstract of each article were independently screened by two of a subset of six authors (LQ, MS, MR, MM, HM, ZT) to ensure relevance and remove duplicates. Any conflicts were discussed amongst the authorship team and resolved by ZT. Full-text articles were then screened via the same process.

For each study, data extraction was conducted by any two authors independently with any conflicts resolved by ZT. Where possible, data extracted from each study were the country where the study was conducted, patient ethnicity, age and gender, and sample size. For COVID-19 infection-related studies, GD onset (time between first COVID-19 symptoms and kidney biopsy), GD diagnosis, kidney biopsy results, GD treatment, GD outcome, interventions, and patient mortality were extracted, where available. For studies relating to COVID-19 vaccinations, dose number, vaccine type, time between vaccinations, GD onset (time between the most recent vaccination and kidney biopsy), GD diagnosis, kidney biopsy results, GD treatment, GD outcome, interventions, and patient mortality were extracted, where available.

### Outcomes of interest and statistical analyses

The primary outcome was to describe the characteristics of patients who developed GD in the setting of COVID-19 infection and vaccination, the relationship between COVID-19 infection and vaccination with GD, the common management strategies, and the overall outcomes of the disease. Descriptive statistics was used to summarise all data collected using frequencies and percentages.

## Results

**Fig. 1:**
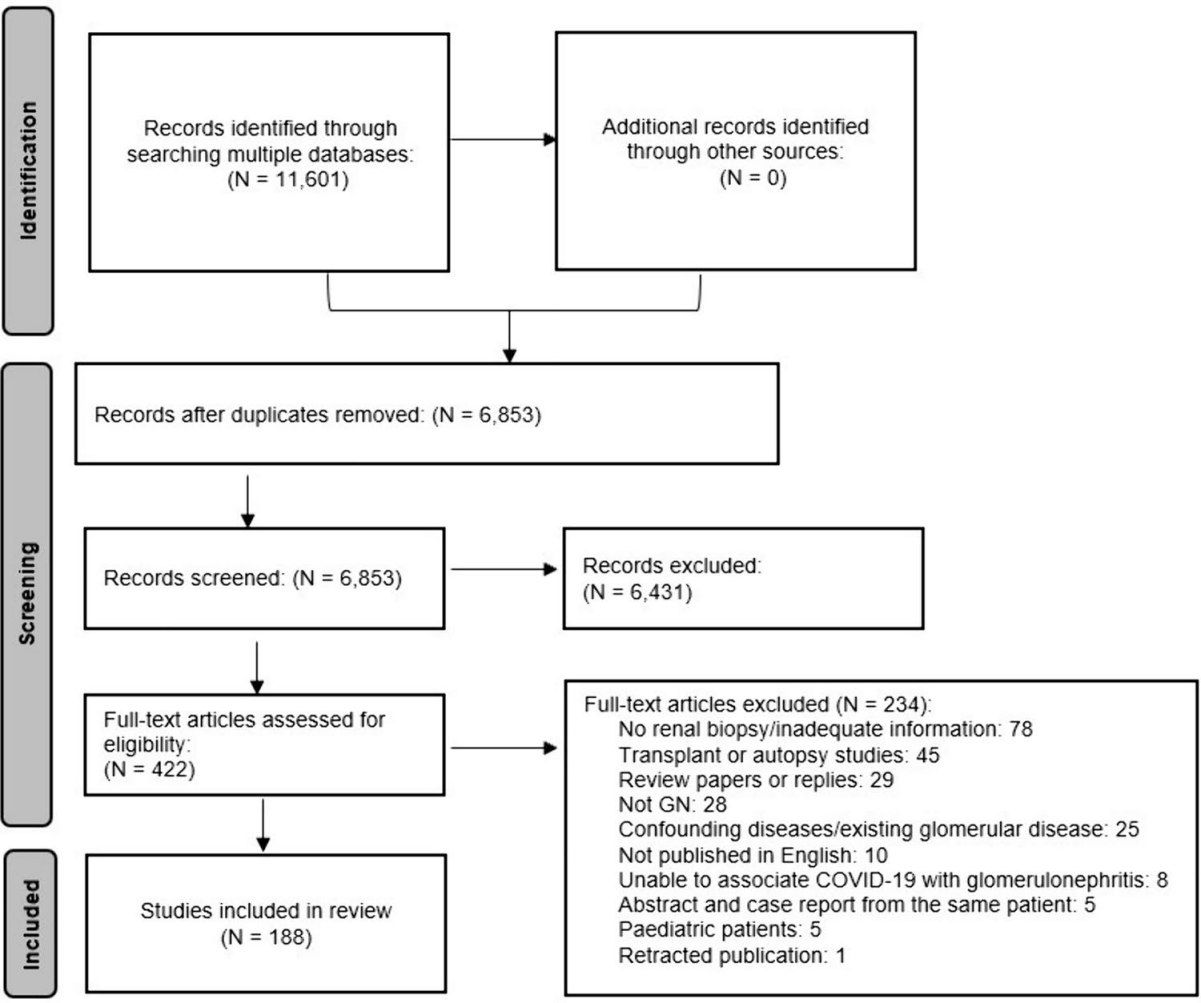
PRISMA flow chart of article identification and inclusion

A flow chart of articles included at each screening phase is presented in Fig. 1. A total of 11,601 studies were retrieved via database searching (Medline = 2831, PubMed = 1059, Scopus = 2678, Embase = 4961, Cochrane = 72) with 4748 duplicates removed. Of the 6853 titles and abstracts screened, 6,431 were deemed irrelevant leaving 422 articles for full-text screening. After excluding 234 full-text articles, 188 remained for inclusion in the review. Of the 188 studies, 106 studies described a total of 341 patients with GD following COVID-19 infection and 82 studies described 146 patients with GD following a COVID-19 vaccination.

### Glomerular disease (GD) following COVID-19 infection

#### All cases of GD

**Fig. 2:**
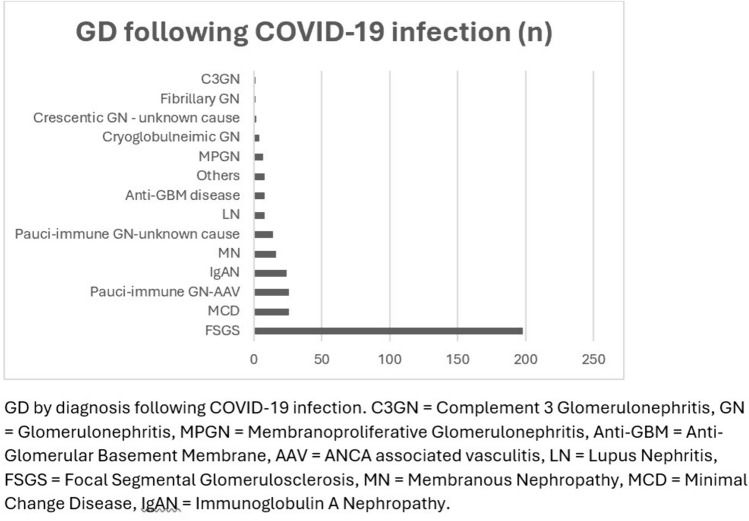
GD by diagnosis following COVID-19 infection

There were 341 cases of GD reported following COVID-19 infection (Fig. 2 and Supplementary Table 2). Patients’ demographics are described in Table 1. Of the available data (approximately 55% of the cohort), the median age was 54 years (IQR 41–63). Data relating to gender were available for 57% (n = 194) of patients and the male-to-female ratio was 2.0:1.0. The majority of reported ethnicities in these patients were African (31%, n = 104) with no data on ethnicity available for 62% (n = 211) of patients. Of the available data (approximately 32% of the cohort), the median GD onset was 17 days (IQR 13–35).

**Table 1:** Demographics of patients with glomerular disease following COVID-19 infection and vaccination

	GD following COVID-19 infection	GD following COVID-19 vaccination
Patients GD following COVID-19 infection/vaccination (n)	341	146
GD types (n)		
FSGS	198	9
MCD	25	36
MN	16	12
IgAN	24	43
MPGN	7	3
LN	8	6
Pauci-immune GN-AAV	26	21
Pauci-immune GN-unknown cause	13	1
Crescentic GN-unknown cause	2	6
Anti-GBM disease	8	6
Cryoglobulinemic GN	4	0
Fibrillary GN	1	1
C3GN	1	0
Others	8	2
Age (years) (median (IQR))	54 (41–63)	52 (34–67)
Sex (n (%))		
Male	130 (38%)	73 (50%)
Female	64 (19%)	67 (46%)
NA	147 (43%)	6 (4%)
Ethnicity (n (%))		
African	104 (31%)	5 (4%)
Caucasian	8 (2%)	45 (31%)
Hispanic/Latino	7 (2%)	8 (5%)
Asian	10 (3%)	10 (7%)
Others	1 (< 1%)	0 (0%)
NA	211 (62%)	78 (53%)
Region (n (%))		
North America	136 (40%)	72 (50%)
South America	1 (< 1%)	6 (4%)
Europe	44 (13%)	24 (16%)
Asia	25 (7%)	42 (29%)
Africa	4 (1%)	0 (0%)
Others	0 (0%)	2 (1%)
Mixed	131 (39%)	0 (0%)
Onset of GD (days) (median (IQR)	17 (13–35)	14 (6–28)
Covid-19 vaccination type (n (%))	NA	
(A) mRNA		113 (78%)
Pfizer		64 (57%)
Moderna		33 (29%)
Unspecified		16 (14%)
(B) Viral vector-based		25 (17%)
AstraZeneca		21 (84%)
Janssen		3 (12%)
Sputnik		1 (4%)
(C) Inactivated whole virus		
		6 (4%)
Sinovac Biotech		4 (66%)
CorV/Sinopharm		1 (17%)
Covaxin		1 (17%)
(D) Recombinant protein subunit		2 (1%)
Covax		2
Vaccination frequency (n (%))		
1		42 (29%)
2		82 (56%)
3		1 (< 1%)
NA		21 (14%)
Immunosuppressive treatment (n, %)		
Yes	110 (32%)	108 (74%)
No	12 (4%)	11 (8%)
NA	219 (64%)	27 (18%)
GD outcomes (n (%))		
Complete remission	21 (6%)	33 (23%)
Partial remission	43 (13%)	29 (20%)
Responding to the treatment	23 (7%)	35 (24%)
No response	28 (8%)	19 (13%)
Not available	226 (66%)	30 (20%)

COVID-19, coronavirus disease 2019; GD, glomerular disease; FSGS, focal segmental glomerulosclerosis; MCD, minimal change disease; IgAN, immunoglobulin A nephropathy; MPGN, membranoproliferative glomerulonephritis; LN, lupus nephritis; GN, glomerulonephritis; AAV, anti-neutrophil cytoplasmic antibodies associated vasculitis; Anti-GBM, anti-glomerular basement membrane; C3GN, complement 3 glomerulonephritis; mRNA, messenger ribonucleic acid; IQR, interquartile range; NA, not applicable/not available

Of all the cases, 27% (n = 92) received immunosuppression for GD, 1% (n = 2) received no immunosuppression, and 72% (n = 247) had no data on immunosuppression. All patients treated with immunosuppression received corticosteroids with 26% (n = 24) receiving cyclophosphamide and 9% (n = 8) receiving rituximab. For the GD outcome, 6% (n = 21) had complete remission, 13% (n = 43) had partial remission, 7% (n = 23) were responding to treatment, and 8% (n = 28) did not respond to treatment.

#### Five most common glomerular diseases following COVID-19 infection

The 5 most common GD following COVID-19 infection were FSGS, anti-neutrophil cytoplasmic antibodies (ANCA) associated vasculitis (AAV), MCD, IgAN, and MN. Details of each GD are described in Table 2.

**Table 2:** Five most common glomerular diseases following COVID-19 infection or vaccination

GD following COVID-19 infection		GD following COVID-19 vaccination	
FSGS (n = 198)		IgAN (n = 43)	
Collapsing	189 (95%)	Crescentic	1 (2%)
Non-collapsing	9 (5%)	Non-crescentic	42 (98%)
Age (years) (median (IQR))	56 (46–63)	Age (years) (median (IQR))	38 (25–62)
Sex (n (%))		Sex (n (%))	
Female	48 (24%)	Female	18 (42%)
Male	68 (34%)	Male	24 (56%)
NA	82 (42%)	NA	1 (2%)
Onset of GD (days) (median (IQR))	14 (13–25)	Onset of GD (days) (median (IQR))	12 (2–24)
Associated with ATN (n (%))		Associated with ATN (n (%))	
Yes	70 (35%)	Yes	8 (19%)
No	19 (10%)	No	20 (47%)
NA	109 (55%)	NA	15 (35%)
Associated with ATIN (n (%))		Associated with ATIN (n (%))	
Yes	51 (26%)	Yes	5 (11%)
No	38 (19%)	No	24 (56%)
NA	109 (55%)	NA	14 (33%)
Associated with TMA (n (%))		Associated with TMA (n (%))	
Yes	7 (4%)	Yes	0 (0%)
No	82 (41%)	No	29 (67%)
NA	109 (55%)	NA	14 (33%)
EM done (n (%))		EM done (n (%))	
Yes	52 (26%)	Yes	18 (42%)
No	0 (0%)	No	1 (2%)
NA	146 (74%)	NA	24 (56%)
Viron or virus like particles (n (%))		NA	NA
Yes	16 (8%)		
No	36 (18%)		
NA	146 (74%)		
Immunosuppressive treatment (n (%))		Immunosuppressive treatment (n (%))	
Yes	32 (16%)	Yes	26 (60%)
No	1 (< 1%)	No	8 (19%)
NA	165 (83%)	NA	9 (21%)
GD outcomes (n (%))		GD outcomes (n (%))	
Complete remission	7 (4%)	Complete remission	8 (19%)
Partial remission	35 (17%)	Partial remission	10 (23%)
Responding to the treatment	7 (4%)	Responding to the treatment	7 (16%)
No response	20 (10%)	No response	9 (21%)
Not available	129 (65%)	Not available	9 (21%)
AAV (n = 26)		MCD (n = 36)	
AAV with MPO or pANCA antibody	17 (65%)	NA	NA
AAV with PR3 or cANCA antibody	7 (27%)		
AAV with dual AAV antibody	1 (4%)		
Unknown AAV type	1 (4%)		
Age (years) (median (IQR))	60 (47–62)	Age (years) (median (IQR))	50 (34–69)
Sex (n (%))		Sex (n (%))	
Female	12 (46%)	Female	11 (31%)
Male	13 (50%)	Male	21 (58%)
NA	1 (4%)	NA	4 (11%)
Onset of GD (days) (median (IQR))	30 (23–62)	Onset of GD (days) (median (IQR))	19 (17–21)
Associated with ATN (n (%))		Associated with ATN (n (%))	
Yes	11 (42%)	Yes	9 (25%)
No	8 (31%)	No	8 (22%)
NA	7 (27%)	NA	19 (53%)
Associated with ATIN (n (%))		Associated with ATIN (n (%))	
Yes	8 (31%)	Yes	1 (3%)
No	11 (42%)	No	17 (47%)
NA	7 (27%)	NA	18 (50%)
Associated with TMA (n (%))		Associated with TMA (n (%))	
Yes	0 (0%)	Yes	0 (0%)
No	19 (73%)	No	18 (50%)
NA	7 (27%)	NA	18 (50%)
EM done (n (%))		EM done (n (%))	
Yes	6 (23%)	Yes	18 (50%)
No	0 (0%)	No	0 (0%)
NA	20 (77%)	NA	18 (50%)
Viron or virus like particles (n (%))		NA	NA
Yes	6 (23%)		
No	20 (77%)		
NA	0 (0%)		
Immunosuppressive treatment (n (%))		Immunosuppressive treatment (n (%))	
Yes			
No	23 (88%)	Yes	28 (78%)
NA	0 (0%)	No	0 (0%)
	2 (12%)	NA	8 (22%)
GD outcomes (n (%))		GD outcomes (n (%))	
Complete remission	5 (19%)	Complete remission	13 (36%)
Partial remission	5 (19%)	Partial remission	6 (17%)
Responding to the treatment	9 (35%)	Responding to the treatment	4 (11%)
No response	1 (4%)	No response	2 (5%)
Not available	6 (23%)	Not available	11 (31%)
MCD (n = 25)		AAV (n = 21)	
NA	NA	AAV with MPO or pANCA antibody	15 (71%)
		AAV with PR3 or cANCA antibody	4 (19%)
		AAV with dual AAV antibody	1 (5%)
		Unknown AAV type	1 (5%)
Age (years) (median (IQR))	48 (33–62)	Age (years) (median (IQR))	66 (57–71)
Sex (n (%))		Sex (n (%))	
Female	5 (20%)	Female	11 (52%)
Male	8 (32%)	Male	10 (48%)
NA	12 (48%)	NA	0 (0%)
Onset of GD (days) (median (IQR))	21 (insufficient data)	Onset of GD (days) (median (IQR))	21 (19–41)
Associated with ATN (n (%))		Associated with ATN (n (%))	
Yes	7 (28%)	Yes	4 (19%)
No	4 (16%)	No	10 (48%)
NA	14 (56%)	NA	7 (33%)
Associated with ATIN (n (%))		Associated with ATIN (n (%))	
Yes	3 (12%)	Yes	3 (15%)
No	8 (32%)	No	11 (52%)
NA	14 (56%)	NA	7 (33%)
Associated with TMA (n (%))		Associated with TMA (n (%))	
Yes	1 (4%)	Yes	0 (0%)
No	10 (40%)	No	14 (67%)
NA	14 (56%)	NA	7 (33%)
EM done (n (%))		EM done (n (%))	
Yes	9 (36%)	Yes	4 (19%)
No	0 (0%)	No	0 (0%)
NA	16 (64%)	NA	17 (81%)
Viron or virus like particles (n (%))		NA	NA
Yes	9 (36%)		
No	0 (0%)		
NA	16 (64%)		
Immunosuppressive treatment (n (%))		Immunosuppressive treatment (n (%))	
Yes	7 (28%)	Yes	21 (100%)
No	0 (0%)	No	0 (0%)
NA	18 (72%)	NA	0 (0%)
GD outcomes (n (%))		GD outcomes (n (%))	
Complete remission	5 (20%)	Complete remission	5 (24%)
Partial remission	0 (0%)	Partial remission	3 (14%)
Responding to the treatment	0 (0%)	Responding to the treatment	10 (48%)
No response	3 (12%)	No response	0 (0%)
Not available	17 (68%)	Not available	3 (14%)
IgAN (n = 24)		MN (n = 12)	
Crescentic	4 (17%)	PLA2R (+)	3 (25%)
Non-crescentic	20 (83%)	PLA2R (−)	0
		THSD7A (+)	1 (8%)
		NELL1	2 (17%)
		Antigen type unknown	6 (50%)
Age (years) (median (IQR))	46 (21–72)	Age (years) (median (IQR))	50 (47–56)
Sex (n (%))		Sex (n (%))	
Female	1 (4%)	Female	3 (25%)
Male	13 (54%)	Male	8 (67%)
NA	10 (42%)	NA	1 (8%)
Onset of GD (days) (median (IQR))	39 (16–105)	Onset of GD (days) (median (IQR))	27 (6–30)
Associated with ATN (n (%))		Associated with ATN (n (%))	
Yes	3 (13%)	Yes	3 (25%)
No	10 (41%)	No	4 (33%)
NA	11 (46%)	NA	5 (42%)
Associated with ATIN (n (%))		Associated with ATIN (n (%))	
Yes	4 (17%)	Yes	1 (8%)
No	9 (37%)	No	6 (50%)
NA	11 (46%)	NA	5 (42%)
Associated with TMA (n (%))		Associated with TMA (n (%))	
Yes	1 (4%)	Yes	0 (0%)
No	12 (50%)	No	7 (58%)
NA	11 (46%)	NA	5 (42%)
EM done (n (%))		EM done (n (%))	
Yes	7 (29%)	Yes	4 (33%)
No	0 (0%)	No	0 (0%)
NA	17 (71%)	NA	8 (67%)
Viron or virus like particles (n (%))		NA	NA
Yes	1 (4%)		
No	6 (25%)		
NA	17 (71%)		
Immunosuppressive treatment (n (%))		Immunosuppressive treatment (n (%))	
Yes	10 (42%)	Yes	3 (25%)
No	0 (0%)	No	3 (25%)
NA	14 (58%)	NA	6 (50%)
GD outcomes (n (%))		GD outcomes (n (%))	
Complete remission	3 (13%)	Complete remission	2 (17%)
Partial remission	0 (0%)	Partial remission	5 (41%)
Responding to the treatment	3 (13%)	Responding to the treatment	2 (17%)
No response	2 (8%)	No response	2 (17%)
Not available	16 (66%)	Not available	1 (8%)
MN (n = 16)		FSGS (n = 9)	
PLA2R (+)	2 (13%)	Collapsing	5 (56%)
PLA2R (−)	1 (6%)	Non-collapsing	4 (44%)
THSD7A (+)	0 (0%)		
NELL1	0 (0%)		
Antigen type unknown	13 (81%)		
Age (years) (median (IQR))	Insufficient data	Age (years) (median (IQR))	47 (30–63)
Sex (n (%))		Sex (n (%))	
Female	3 (19%)	Female	5 (56%)
Male	0 (0%)	Male	4 (44%)
NA	13 (81%)	NA	0 (0%)
Onset of GD (days) (median (IQR))	NA	Onset of GD (days) (median (IQR))	21 (10–37)
Associated with ATN (n (%))		Associated with ATN (n (%))	
Yes	2 (12%)	Yes	4 (45%)
No	1 (6%)	No	1 (10%)
NA	13 (81%)	NA	4 (45%)
Associated with ATIN (n (%))		Associated with ATIN (n (%))	
Yes	2 (12%)	Yes	0 (0%)
No	1 (6%)	No	5 (56%)
NA	13 (82%)	NA	4 (44%)
Associated with TMA (n (%))		Associated with TMA (n (%))	
Yes	0 (0%)	Yes	0 (0%)
No	3 (19%)	No	5 (56%)
NA	13 (81%)	NA	4 (44%)
EM done (n (%))		EM done (n (%))	
Yes	3 (19%)	Yes	5 (56%)
No	0 (0%)	No	0 (0%)
NA	13 (81%)	NA	4 (44%)
Viron or virus like particles (n (%))		NA	NA
Yes	0 (0%)		
No	3 (19%)		
NA	13 (81%)		
Immunosuppressive treatment (n (%))		Immunosuppressive treatment (n (%)) Yes	
Yes	1 (6%)	No	7 (78%)
No	1 (6%)	NA	0 (0%)
NA	14 (88%)		2 (22%)
GD outcomes (n (%))		GD outcomes (n (%))	
Complete remission	0 (0%)	Complete remission	1 (11%)
Partial remission	1 (6%)	Partial remission	2 (22%)
Responding to the treatment	0 (0%)	Responding to the treatment	3 (34%)
No response	0 (0%)	No response	1 (11%)
Not available	15 (94%)	Not available	2 (22%)

COVID-19, coronavirus disease 2019; GD, glomerular disease; FSGS, focal segmental glomerulosclerosis; IgAN, immunoglobulin A nephropathy; AAV, anti-neutrophil cytoplasmic antibodies associated vasculitis; MCD, minimal change disease; MN, membranous nephropathy; ATN, acute tubular necrosis; ATIN, acute tubulointerstitial necrosis; TMA, thrombotic microangiopathy; EM, electron microscopy; MPO, myeloperoxidase; PR3, proteinase 3; pANCA, perinuclear anti-neutrophil cytoplasmic antibodies; cANCA, cytoplasmic anti-neutrophil cytoplasmic antibodies; PLA2R, M-type phospholipase A2 receptor; THSD7A, thrombospondin type-1 domain-containing 7A; NELL1, neural epidermal growth factor-like 1 protein; NA, not applicable/not available; IQR, interquartile range

#### Focal segmental glomerulosclerosis (FSGS)

FSGS was the most common GD following COVID-19 infection at 198 cases. Of the available data (64% of the cohort), the median age was 56 years (IQR 46–63). Data relating to gender were available for 59% (n = 116) of patients and the male-to-female ratio was 2:1. The majority of reported ethnicities in these patients were African (43%, n = 85) with no data on ethnicity available for 54% (n = 108) of patients. Of available data (34% of the cohort), the median onset of FSGS was 14 days (IQR 13–25).

For treatment, 16% (n = 32) received immunosuppression for FSGS, < 1% (n = 1) received no immunosuppression and 83% (n = 165) had no available data on immunosuppression. In the patients treated with immunosuppression, 91% (n = 29) received corticosteroids, 6% (n = 2) received calcineurin inhibitor (CNI), 3% (n = 1) received mycophenolate (MMF), and 3% (n = 1) received cyclophosphamide. For the FSGS outcome, 4% (n = 7) had complete remission, 17% (n = 35) had partial remission, 4% (n = 7) were responding to the treatment, and 10% (n = 20) did not respond to treatment.

#### ANCA associated vasculitis (AAV)

There were 26 cases of AAV following COVID-19 infection with a median age of 60 years (IQR 47–62), and a male-to-female ratio of 1.1:1.0. The majority of reported ethnicities in these patients were African (12%, n = 3) with no data on ethnicity available for 72% (n = 19) of patients. Of the available data (62% of the cohort), the median onset of AAV was 30 days (IQR 23–62).

Among this cohort, 65% (n = 17) of cases were associated with myeloperoxidase (MPO)-ANCA or perinuclear (P)-ANCA, 27% (n = 7) of cases with proteinase 3 (PR3)-ANCA or cytoplasmic (C)-ANCA, 4% (n = 1) of cases with dual antibodies, and in 4% (n = 1) of cases the type of ANCA was not specified.

For treatment, 88% (n = 23) received immunosuppression for AAV, and 12% (n = 3) had no data on immunosuppression. All patients treated with immunosuppression received corticosteroids with 27% (n = 7) receiving rituximab, 52% (n = 12) receiving cyclophosphamide, and 22% (n = 5) receiving plasma exchange. For the AAV outcome, 35% (n = 9) were responding to the treatment, 19% (n = 5) had complete remission, 19% (n = 5) had partial remission, and 4% (n = 1) did not respond to treatment.

#### Minimal change disease (MCD)

There were 25 cases of MCD following COVID-19 infection. Of the available data (52% of the cohort), the median age was 48 years (IQR 33–62), and the male-to-female ratio was 1.6:1.0. The majority of reported ethnicities in these patients were African (16%, n = 4) with no data on ethnicity available for 68% (n = 17) of patients. Of the available data (24% of the cohort), the median onset of MCD was 21 days (IQR insufficient data).

For treatment, 28% (n = 7) received immunosuppression for GD, and 72% (n = 18) had no data on immunosuppression. All patients treated with immunosuppression received corticosteroids. For the GD outcome, 20% (n = 5) had complete remission, and 12% (n = 3) did not respond to treatment.

#### IgA nephropathy (IgAN*)*

There were 24 cases (20 non-crescentic and 4 crescentic) of IgAN following COVID-19 infection. Of the available data (67% of the cohort), the median age was 46 years (IQR 21–72). Data relating to gender were available for 58% (n = 14) of patients and the male-to-female ratio was 12.0:1.0. The majority of reported ethnicities in these patients were Caucasian (8%, n = 2) with no data on ethnicity available for 88% (n = 21) of patients. Of the available data (29% of the cohort), the median onset of IgAN was 39 days (IQR 16–105).

For treatment, 42% (n = 10) received immunosuppression for IgAN, and 58% (n = 14) had no data on immunosuppression. All patients treated with immunosuppression received corticosteroids. For the IgAN outcome, 13% (n = 3) had complete remission, 13% (n = 3) were responding to the treatment, and 8% (n = 2) did not respond to treatment.

#### Membranous nephropathy (MN)

There were 16 cases of MN following COVID-19 infection (13 cases with unspecified antibody, 2 cases with anti-PLA2R positive, 1 case with anti-PLA2R negative). In 81% (n = 13) of patients there were no data on the age, gender, ethnicity, and MN onset and 94% (n = 15) had no data on GD treatment outcome.

### Glomerular disease (GD) following COVID-19 vaccinations

#### All cases of GD

**Fig. 3:**
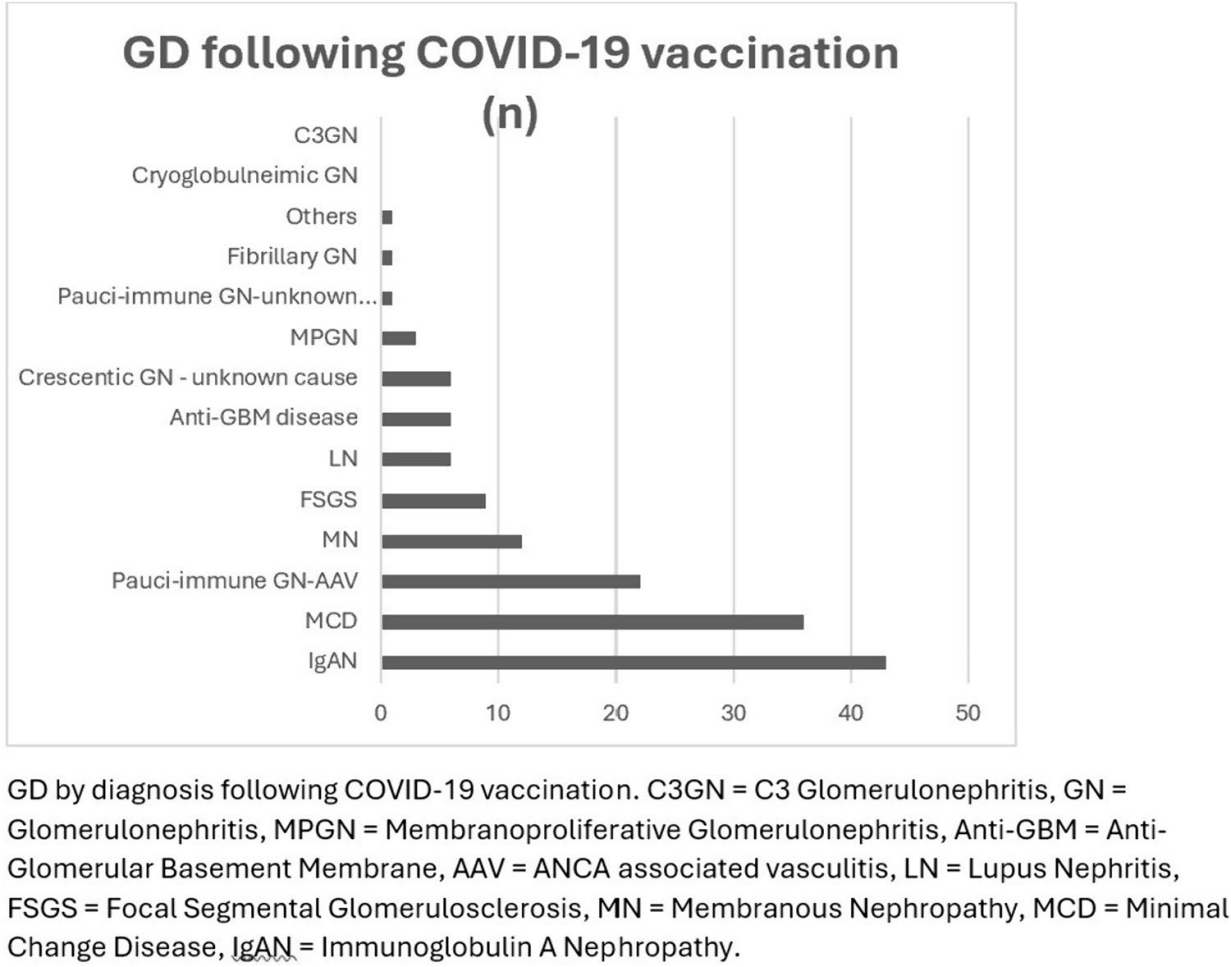
GD by diagnosis following COVID-19 vaccination

There were 146 reported cases of GD following a COVID-19 vaccine (Fig. 3 and Supplementary Table 3). Patients’ demographics are described in Table 1. Of the available data (97% of the cohort), the median age was 52 years (IQR 34–67), and the male-to-female ratio was 1.1:1.0. The majority of reported ethnicities in these patients were Caucasian (31%, n = 45) with no data on ethnicity available for 53% (n = 78) of patients. Of the available data (90% of the cohort), the median onset of GD with a renal biopsy was 14 days (IQR 6–28).

**Fig. 4:**
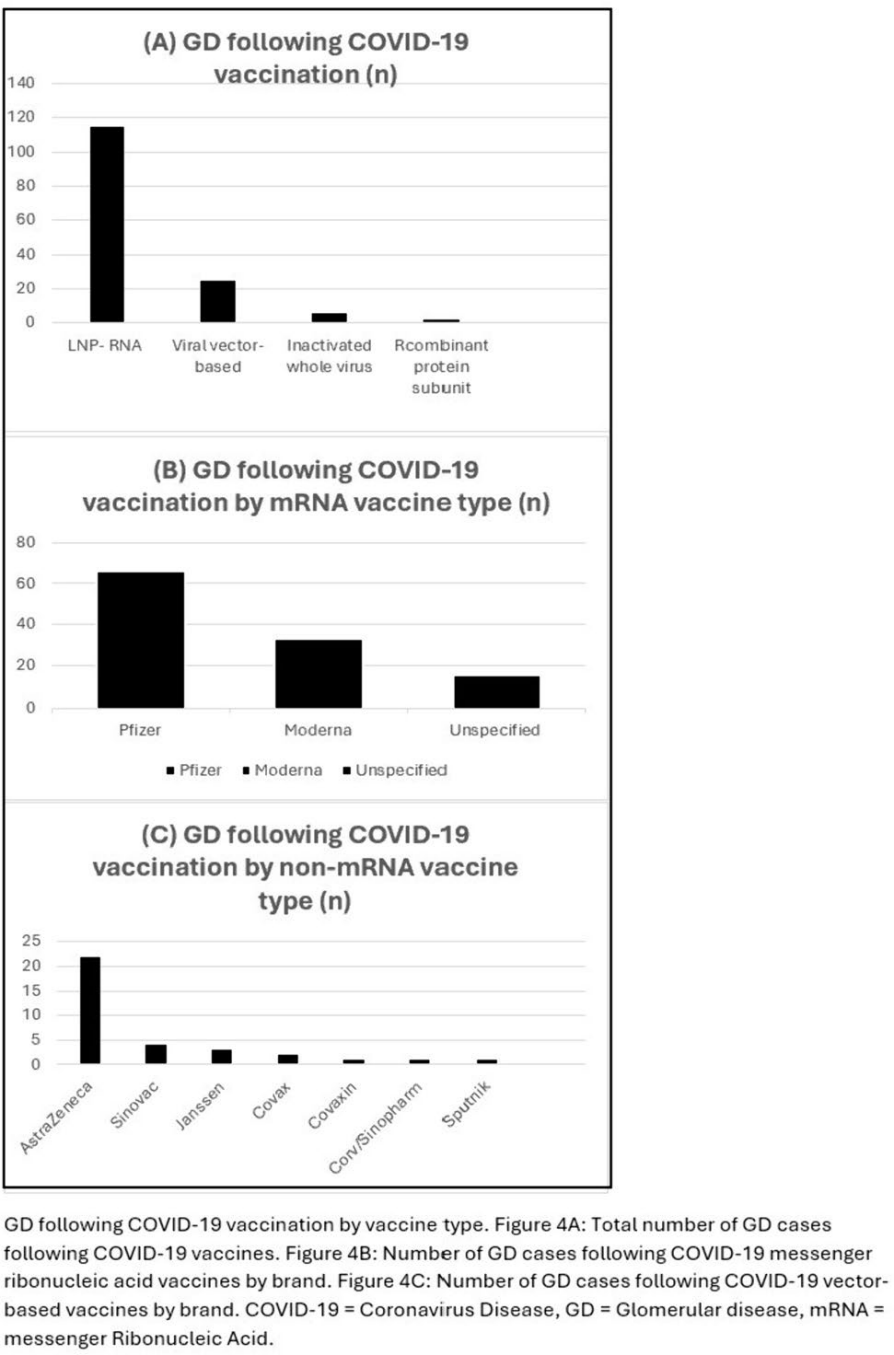
GD following COVID-19 vaccination by vaccine type

Of the GD associated with COVID-19 vaccinations, 78% (n = 113) developed following mRNA vaccines (Moderna 33, Pfizer 64, unspecified 16), 17% (n = 25) developed following viral vector-based vaccines (AstraZeneca 21, Janssen 3, Sputnik 1), 4% (n = 6) developed following inactivated whole virus vaccines (Covaxin 1, Sinopharm 1, Sinovac 4), and 1% (n = 2) developed following recombinant protein vaccines (Covax 2) (Fig. 4). In terms of timing, 29% (n = 42) developed GD after the first vaccination, 56% (n = 82) after the second vaccination, and < 1% (n = 1) after the third vaccination. For treatment, 74% (n = 108) of patients received immunosuppression for GD, 8% (n = 11) received no immunosuppression, and 18% (n = 27) had no data on immunosuppression. In the patients treated with immunosuppression, 96% (n = 104) received corticosteroids, 26% (n = 28) received cyclophosphamide, 10% (n = 11) received rituximab, and 7% (n = 8) received MMF. For the reported GD outcomes, 23% (n = 33) had complete remission, 20% (n = 29) had partial remission, 24% (n = 35) were responding to treatment, and 13% (n = 19) did not respond to treatment.

#### Five most common glomerular diseases following COVID-19 vaccination

The five most common GDs following COVID-19 vaccinations were IgAN, MCD, AAV, MN, and FSGS. Details of each GD are described in Table 2.

#### IgA nephropathy (IgAN)

IgAN was the most common GD following COVID-19 vaccination at 43 cases (42 non-crescentic and 1 crescentic). Of the available data (98% of the cohort), the median age was 38 years (IQR 25–62), and the male-to-female ratio was 1.3:1.0. The majority of reported ethnicities in these patients were Caucasian (42%, n = 18) with no data on ethnicity available for 41% (n = 17) of patients. Of the available data (approximately 84% of the cohort), the median onset of IgAN was 12 days (IQR 2–24).

In terms of vaccination type, 90% (n = 39) of cases developed following mRNA vaccines (Moderna 9, Pfizer 23, unspecified 7), 5% (n = 2) developed following viral vector-based vaccines (AstraZeneca 2), and 5% (n = 2) developed following inactivated whole virus vaccines (Sinopharm 1, Sinovac 1). Most patients developed IgAN following the second vaccination at 75% (n = 32) with 16% (n = 7) of patients developing it after the first vaccination. For treatment, 60% (n = 26) received immunosuppression for IgAN, 19% (n = 8) received no immunosuppression, and 21% (n = 9) had no data on immunosuppression. All patients treated with immunosuppression received corticosteroids with 19% (n = 5) receiving cyclophosphamide and 8% (n = 2) receiving MMF. For the IgAN outcome, 19% (n = 8) had complete remission, 23% (n = 10) had partial remission, 16% (n = 7) were responding to treatment, and 21% (n = 9) did not respond to treatment.

#### Minimal change disease (MCD)

There were 36 cases of MCD following COVID-19 vaccinations. Of the available data (89% of the cohort), the median age was 50 years (IQR 34 -69), and the male-to-female ratio was 1.9:1.0. The majority of reported ethnicities in these patients were Caucasian (25%, n = 9) with no data on ethnicity available for 66% (n = 24) of patients. The median onset of MCD was 19 days (IQR 7–21).

There were 77% (n = 28) of cases reported following mRNA vaccines (Moderna 7, Pfizer 15, unspecified 6), 17% (n = 6) of cases following viral vector-based vaccines (AstraZeneca 5, Janssen 1), and 6% (n = 2) of cases following inactivated whole virus vaccines (Sinovac 2). Most patients developed MCD after the first vaccination at 47% (n = 17) with 39% (n = 14) developing MCD after the second vaccination. For treatment, 78% (n = 28) received immunosuppression for MCD and 22% (n = 8) had no data on immunosuppression. All patients treated with immunosuppression received corticosteroids and 4% (n = 1) received cyclophosphamide. For the GD outcome, 36% (n = 13) had complete remission, 17% (n = 6) had partial remission, 11% (n = 4) were responding to treatment, and 5% (n = 2) did not respond to treatment.

#### ANCA associated vasculitis (AAV)

There were 21 cases of AAV following COVID-19 vaccinations. The median age was 66 years (IQR 57–71), with a male-to-female ratio of 1.0:1.1. The most reported ethnicity in these patients was Caucasian (33%, n = 7) with no data on ethnicity available for 57% (n = 12) of patients. The median onset of AAV was 21 days (IQR 19–41).

Among the cohort, 71% (n = 15) of cases were associated with myeloperoxidase (MPO)-ANCA or perinuclear (P)-ANCA, 19% (n = 4) of cases with proteinase 3 (PR3)-ANCA or cytoplasmic (C)-ANCA, 5% (n = 1) of cases with dual antibodies, and in 5% (n = 1) of cases the type of ANCA was not specified.

There were 66% (n = 14) of AAV cases reported after mRNA vaccines (Moderna 6, Pfizer 8), 19% (n = 4) of cases following viral vector-based vaccines (AstraZeneca 3, Janssen 1), 10% (n = 2) of cases following inactivated whole virus vaccines (Covaxin 1, Sinovac 1), and 1 (5%) case following a recombinant protein-based vaccine (Covax 1). In the patients who developed AAV following COVID-19 vaccinations, 29% (n = 6) developed GD after the first vaccination, 52% (n = 11) after the second vaccination, and 5% (n = 1) after the third vaccination. All patients received immunosuppression to treat AAV following COVID-19 vaccination. All patients treated with immunosuppression received corticosteroids, 29% (n = 6) received rituximab, and 48% (n = 10) received cyclophosphamide. Plasma exchange was provided in 19% (n = 4) of cases. For the AAV outcome, 24% (n = 5) had complete remission, 14% (n = 3) had partial remission, and 48% (n = 10) were responding to treatment.

#### Membranous nephropathy (MN)

There were 12 cases of MN reported following COVID-19 vaccinations (6 cases with unspecified antibodies, 3 cases with anti-PLA2R, 1 case with anti-THSD7A and 2 cases with anti-NELL1 antibodies). Of the available data (92% of the cohort), the median age was 50 years (IQR 47–56), with a male-to-female ratio of 2.7:1.0. The most reported ethnicity in these patients was Caucasian (25%, n = 3) with no data on ethnicity available for 58% (n = 7) of patients. The median onset of MN was 27 days (IQR 6–30).

There were 75% (n = 9) of cases reported following mRNA vaccines (Moderna 3, Pfizer 4, unspecified 2), and 25% (n = 3) of cases following viral vector-based vaccines (AstraZeneca 2, Janssen 1). In the patients who developed MN following COVID-19 vaccinations, 16% (n = 2) developed GD after the first vaccination and 42% (n = 5) after the second vaccination. Only 25% (n = 3) received immunosuppression for MN with 25% (n = 3) receiving no immunosuppression, and 50% (n = 6) of patients had no data on immunosuppression. All patients treated with immunosuppression received corticosteroids, 33% (n = 1) received rituximab, and 66% (n = 2) received cyclophosphamide. For the MN outcome, 17% (n = 2) had complete remission, 41% (n = 5) had partial remission, 17% (n = 2) were responding to treatment, and 17% (n = 2) did not respond to treatment.

#### Focal segmental glomerulosclerosis (FSGS)

There were 9 cases of FSGS following COVID-19 vaccinations (5 collapsing, and 4 non-collapsing FSGS), including 1 case with a mix of collapsing glomerulopathy and membranous nephropathy post-vaccination. The median age was 47 years (IQR 30–63), with a male-to-female ratio of 1.0:1.3. The most reported ethnicity in these patients was Caucasian (23%, n = 2) with no data on ethnicity available for 33% (n = 3) of patients. The median onset of FSGS was 21 days (IQR 10–37).

There were 67% (n = 6) of cases reported following mRNA vaccines (Moderna 3, Pfizer 3), and 33% (n = 3) of cases following viral vector-based vaccines (AstraZeneca 3). In the patients who developed FSGS following COVID-19 vaccinations, 33% (n = 3) developed GD after the first vaccination and 67% (n = 6) after the second vaccination. For treatment, 78% (n = 7) received immunosuppression. and 22% (n = 2) had no data on immunosuppression. All patients treated with immunosuppression received corticosteroids and 14% (n = 1) received cyclophosphamide. For the FSGS outcome, 11% (n = 1) had complete remission, 22% (n = 2) had partial remission, 34% (n = 3) were responding to treatment, and 11% (n = 1) did not respond to treatment.

### Comparison between GD associated with COVID-19 infection and vaccination

**Fig. 5:**
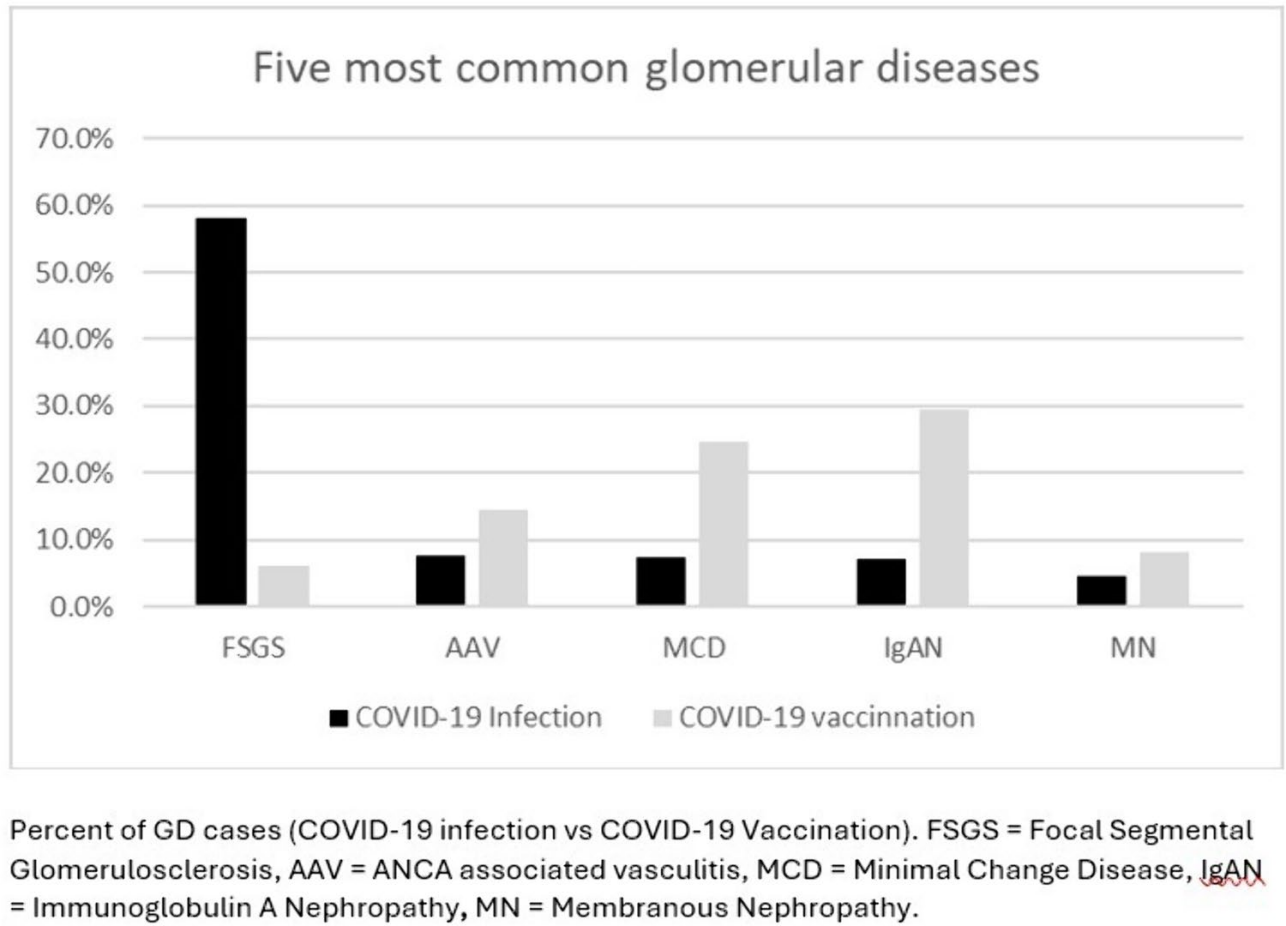
Percent of GD cases (COVID-19 infection vs COVID-19 Vaccination)

As described in Fig. 5, 58% of patients developed FSGS with the infection compared to 6% with vaccination, and 8% of patients developed IgAN with the infection compared to 29% with vaccination. The rates of other common GDs are MCD (7% vs 25%), AAV (8% vs 14%), and MN (5% vs 9%). Overall, 15% (n = 22) of the vaccine cohort and 32% (n = 110) infection cohort required treatment with dialysis.

## Discussion

This scoping review describes de novo GD cases following COVID-19 infection and vaccination. Overall, the number of GD cases was relatively low given the large number of COVID-19 infections and administered vaccinations. Frequently reported GD were FSGS (specifically collapsing glomerulopathy), IgAN, MCD, AAV, and MN in both infection and vaccination cohorts.

Vaccines have been known to be associated with GD with onset observed in patients after immunisation against influenza, pneumococcus, and hepatitis B [16]. GD was more likely to develop after receiving mRNA vaccinations or viral-vectored vaccines when compared to the other types of vaccinations. A possible explanation could be that both mRNA vaccines (Pfizer-BioNTech and Moderna) and viral-vectored vaccines (AstraZeneca and Janssen) induce balanced humoral and T cell immunity compared with inactivated whole virus vaccines (Sinovac Biotech) which may precipitate immune-mediated GD [17]. The most common GD following COVID-19 vaccination was IgAN and was most frequently reported in the Caucasian population. This was unexpected as typically Asian ethnicity is associated with IgAN [18]. This could perhaps be explained by the increased case reports published by countries with a higher Caucasian population or the large proportion of ethnicity data that was not reported (53% not reported in the vaccination cohort).

In the infection cohort, the most reported GD was FSGS and was most frequently reported in patients of African ethnicity. This was not unexpected as an association has been previously described between COVID-19 infection causing collapsing glomerulopathy in patients with the high-risk Apolipoprotein L gene 1 (APOL1) polymorphisms, often found in patients of African ethnicity [7]. The association of APOL1 polymorphism and collapsing glomerulopathy was also previously reported in patients with other viral infections [19].

GD is thought to develop following COVID-19 infection with the SARS-Cov-2 virus, an enveloped virus containing a single-stranded RNA genome, binding to host angiotensin-converting enzyme 2 (ACE2) receptors through spike S-glycoproteins to mediate fusion and viral penetration and subsequent immune response [1]. There have been more reports of other glomerular pathology associated with COVID-19 besides collapsing glomerulopathy [19]. In our report, other commonly reported GD following COVID-19 infection were AAV, MCD, IgAN, and MN. The exact mechanism of how these diseases occur is still unclear; however, some hypothesise a direct viral involvement affecting the glomerulus and others suggest a systemic immunological response to the virus to trigger an immune-mediated glomerulonephritis [20].

Patients were more likely to be treated with immunosuppression in the vaccine cohort (74%, n = 111) compared to the infection cohort (33%, n = 112). This may be due to the challenge of treating GD in patients with active COVID-19 infection. Corticosteroid use was discouraged early in the pandemic as it was thought to suppress the immune system, however, after several randomised control trials, corticosteroids have been shown to suppress the lethal cytokine storm associated with severe COVID-19 and improve patient survival [21]. To date, there are no clear guidelines on how to manage these patients [22].

In the infection cohort, 6% (n = 19) had complete remission, 13% (n = 45) partial remission, and 7% (n = 23) were responding to the treatment. In the vaccine cohort, 21% (n = 32) had complete remission, 20% (n = 30) had partial remission, and 23% (n = 35) were responding to the treatment. There was significant morbidity described in both cohorts as 15% (n = 22) of the vaccine cohort and 32% (n = 110) infection cohort required dialysis.

The limitation of this scoping review is that it likely underestimates the number of GD cases following COVID-19 infection and vaccination for multiple reasons. Not every patient would have been published as a case report or case series meaning that the cases not published could not have been included in this review. In most cases, the diagnosis of GD requires a renal biopsy. Unfortunately, GD may be underdiagnosed as not all patients with AKI, proteinuria and/or haematuria in the setting of COVID-19 infection or vaccination would have been biopsied. In addition, caution should be taken when drawing conclusions from small sample sizes and descriptive studies, with articles included in this review mostly comprised of case reports and case series. With some of the larger studies, substantial data was missing making it possible that some trends may not truly reflect population data.

## Future directions

More research into specific pathophysiology is needed to better understand the relationship between COVID-19 infections, vaccinations, and GD. Larger population-based studies would assist in determining changes in the epidemiology of GD with COVID-19 infection and vaccination pre- and post-pandemic eras. Clinicians should be encouraged to report on long-term outcomes of GD and diagnostic and therapeutic approaches to provide support for clinical decision-making revolving around COVID-19-associated GD. Quantitative and qualitative studies on the experience of clinicians in managing GD patients during the COVID-19 pandemic will provide further insight into this challenging area and may identify future research agendas. Larger prospective studies should be designed to address the current knowledge gap around this challenging condition when patients develop new infections or receive booster vaccinations.

## Conclusion

This scoping review describes a substantial number of GD cases following COVID-19 infection and vaccination with FSGS most common post-COVID-19 infection and IgAN most common following COVID-19 vaccination, especially the mRNA vaccine. Patients with GD following COVID-19 vaccination were more likely to be treated with immunosuppression with an overall better remission rate compared to the patients who developed GD post-COVID-19 infection. Since the COVID-19 pandemic, there have been increasing reports of GD following COVID-19 infection and vaccination. Although a causal relationship between COVID-19 and GD has not been established, proposed pathogenic mechanisms are plausible. The study designs included in our review are largely case reports, and there needs to be better awareness and reporting of GD in the setting of COVID-19 infection and vaccination to treat and prevent renal disease and improve long-term patient outcomes. Clinicians should be vigilant about this possible association and collaborative research projects are required to fill the epidemiology, pathophysiology, diagnosis, treatment, and prognosis gap.

## Supplementary Information

Below is the link to the electronic supplementary material.

Supplementary file1 (DOCX 213 kb)

Supplementary file2 (DOCX 84 kb)

Supplementary file3 (DOCX 26 kb)

## Data Availability

No datasets were generated or analysed during the current study.
