# Supplementary material for: A scoping review on adult patients with de novo glomerular diseases following COVID-19 infection or vaccine
Source: Int Urol Nephrol. 2024 Sep 3;57(2):447–62. doi: 10.1007/s11255-024-04189-0 (PMC11772384; doi:10.1007/s11255-024-04189-0)
Supplement: Supplementary file 1 — Supplementary file1 (DOCX 213 kb) [file 11255_2024_4189_MOESM1_ESM.docx]

Supplementary table 1: search strategy for each database

| **Database** | **COVID-related search string** | **Glomerular disease-related search string** |
| --- | --- | --- |
| Medline | TX covid* OR TX coronavirus OR TX SARS?CoV?2* | TX "glomerular disease" OR TX glomerulopathy OR TX glomerulonephritis OR TX glomerulosclerosis OR TX proteinuria OR TX h?maturia OR TX "acute kidney injur*" OR TX AKI |
| PubMed | ((covid*[MeSH Terms]) OR (coronavirus[MeSH Terms])) OR (SARS-CoV-2*[Title/Abstract]) | ((((((((glomerular disease[Title/Abstract]) OR (glomerulopathy[Title/Abstract])) OR (glomerulonephritis[MeSH Terms])) OR (glomerulosclerosis[Title/Abstract])) OR (proteinuria[MeSH Terms])) OR (haematuria*[MeSH Terms])) OR (hematuria*[MeSH Terms])) OR (acute kidney injur*[MeSH Terms])) OR (aki[Title/Abstract]) |
| Scopus | (ALL ( covid* ) OR ALL ( coronavirus ) OR ALL ( sars*cov*2* ) ) | ( TITLE-ABS-KEY ( "glomerular disease" )  OR  TITLE-ABS-KEY ( glomerulopathy )  OR  TITLE-ABS-KEY ( glomerulonephritis )  OR  TITLE-ABS-KEY ( glomerulosclerosis )  OR  TITLE-ABS-KEY ( proteinuria )  OR  TITLE-ABS-KEY ( haematuria )  OR  TITLE-ABS-KEY ( hematuria )  OR  TITLE-ABS-KEY ( "acute kidney injur*" )  OR  TITLE-ABS-KEY ( aki ) ) |
| Embase | (covid* OR 'coronavirus'/exp OR sars?cov?2*) | 'glomerulopathy':ab,kw,ti OR 'glomerulonephritis'/exp OR 'glomerulonephritis' OR 'glomerulosclerosis':ab,kw,ti OR 'proteinuria'/exp OR 'proteinuria' OR 'hematuria'/exp OR 'hematuria' OR 'acute kidney failure'/exp OR 'acute kidney failure' OR 'aki':ab,kw,ti |
| Cochrane | ((COVID*) OR (coronavirus) OR (SARS?CoV?2*)) | "glomerular disease" OR glomerulopathy OR glomerulonephritis OR glomerulosclerosis OR proteinuria OR haematuria OR haematuria OR '"acute kidney injur*" OR AKI |

**Supplementary table 2:** Glomerular diseases following COVID-19 infection

| **Author, year** | **Ethnicity** | **Region** | **Age**  **(Years)** | **Sex** | **Onset**  **(Days)** | **GD diagnosis** | **EM done** | **ATN** | **ATIN** | **TMA** | **Virion or virus like particles** | **Immunosuppressive treatment for GD** | **Treatment outcome** |
| --- | --- | --- | --- | --- | --- | --- | --- | --- | --- | --- | --- | --- | --- |
| Adapa et al., 2021^1^ | African | North America | NA | F | NA | FSGS-collapsing glomerulopathy | Yes | NA | NA | NA | No | NA | NA |
| Afonso et al., 2021^2^ | African | Europe | 43 | F | NA | FSGS-non-collapsing-tip variant | NA | NA | NA | NA | NA | NA | Partial remission |
| Akilesh et al., 2021^3^ | African | North America | 60 | F | NA | FSGS-collapsing glomerulopathy | NA | Yes | No | No | NA | NA | NA |
|  | African | North America | 63 | F | NA | FSGS-collapsing glomerulopathy | NA | Yes | No | Yes | NA | NA | NA |
|  | African | North America | 58 | F | NA | FSGS-collapsing glomerulopathy | NA | Yes | Yes | No | NA | NA | Partial remission |
|  | Hispanic | North America | 77 | F | NA | FSGS-non-collapsing glomerulopathy | NA | Yes | No | Yes | NA | NA | NA |
|  | Caucasian | North America | 52 | F | NA | MCD | NA | NA | NA | NA | NA | Yes | Complete remission |
|  | African | North America | 58 | M | NA | FSGS-collapsing glomerulopathy | NA | Yes | No | Yes | NA | NA | Complete remission |
|  | African | North America | 46 | M | NA | FSGS-collapsing glomerulopathy | NA | Yes | No | No | NA | NA | NA |
|  | African | North America | 47 | M | NA | FSGS-collapsing glomerulopathy | NA | Yes | No | Yes | NA | NA | NA |
|  | Caucasian | North America | 44 | M | NA | FSGS-collapsing glomerulopathy | NA | Yes | Yes | No | NA | NA | NA |
|  | African | North America | 59 | M | NA | FSGS-non-collapsing glomerulopathy | NA | Yes | Yes | No | NA | NA | NA |
| Akrawi et al., 2021^4^ | African | North America | 48 | F | 42 | FSGS-collapsing glomerulopathy | NA | NA | NA | NA | NA | Yes | NA |
| Alawad et al., 2022^5^ | Asian | Asia | 27 | M | 3 | Pauci-immune GN- unknown cause | NA | NA | NA | NA | NA | Yes | NA |
| Allena et al., 2021^6^ | NA | North America | 60 | F | 30 | AAV-MPO | NA | Yes | Yes | No | NA | Yes | Responding to the treatment |
| Alotaibi et al., 2022^7^ | NA | North America | 52 | M | 14 | FSGS-collapsing glomerulopathy + granulomatous AIN | Yes | Yes | Yes | No | No | Yes | Partial remission |
| Amin et al., 2022^8^ | NA | Asia | 33 | M | 28 | IgAN | Yes | No | Yes | No | No | NA | NA |
| Apaydin et al., 2021^9^ | NA | Asia | 18 | M | 23 | IgAN-AAV-PR3 positive | NA | No | Yes | No | NA | Yes | No response |
| Asma et al., 2022^10^ | NA | Africa | 72 | M | 60 | AAV-PR3 | Yes | No | Yes | No | No | Yes | Responding to the treatment |
| Basiratnia et al., 2021^11^ | NA | Asia | 17 | M | 14 | Crescentic GN- unknown cause | Yes | Yes | Yes | No | Yes | Yes | No response |
|  | NA | Asia | 16 | M | 5 | Crescentic GN- unknown cause | NA | NA | NA | NA | No | Yes | Partial remission |
| Borrero-Arvelo et al., 2021^12^ | NA | North America | 51 | M | NA | IgAN | Yes | No | No | No | No | NA | NA |
| Boudhabhay et al., 2021^13^ | African | Europe | 46 | M | 7 | Atypical HUS | NA | Yes | Yes | Yes | No | Yes | Complete remission |
| Bugdayci Uner et al., 2021^14^ | NA | Asia | Mean 64 | NA | NA | AAV - ANCA type unknown | NA | NA | NA | NA | NA | NA | NA |
|  | NA | Asia | Mean 64 | NA | NA | Anti-GBM disease | NA | NA | NA | NA | NA | NA | NA |
|  | NA | Asia | Mean 64 | NA | NA | IgAN | NA | NA | NA | NA | NA | NA | NA |
|  | NA | Asia | Mean 64 | NA | NA | IgAN | NA | NA | NA | NA | NA | NA | NA |
|  | NA | Asia | Mean 64 | NA | NA | IgG-kappa and C3GN | NA | NA | NA | NA | NA | NA | NA |
| Chandler et al., 2021^15^ | NA | North America | 48 | F | 60 | AAV-pANCA | NA | No | No | No | NA | NA | Partial remission |
| Chang et al., 2021^16^ | NA | North America | 62 | M | NA | FSGS-collapsing glomerulopathy | NA | NA | NA | NA | NA | Yes | Partial remission |
|  | NA | North America | 63 | M | NA | FSGS-collapsing glomerulopathy | NA | NA | NA | NA | NA | NA | Partial remission |
| Chargui et al., 2021^17^ | NA | Africa | 49 | M | 14 | AAV-MPO | NA | No | No | No | NA | Yes | NA |
| Christodoulou et al., 2022^18^ | Caucasian | Europe | 72 | F | 15 | AAV-MPO | NA | No | No | No | NA | Yes | Complete remission |
| D'Ambrosio et al., 2022^19^ | NA | Europe | 78 | F | NA | AAV-pANCA | NA | NA | NA | NA | NA | Yes | NA |
| Danis et al., 2022^20^ | NA | Asia | 59 | M | 60 | RPGN - Immune mediated GN | Yes | No | Yes | No | Yes | Yes | Partial remission |
| Das et al., 2021^21^ | NA | North America | 63 | F | NA | FSGS-collapsing glomerulopathy | Yes | Yes | No | No | No | Yes | Responding to the treatment |
| DeFabritiis et al., 2021^22^ | NA | Europe | 35 | M | 49 | MCD | Yes | No | Yes | Yes | No | Yes | Complete remission |
| Deshmukh et al., 2020^23^ | Asian | North America | 42 | M | 90 | FSGS-collapsing glomerulopathy | Yes | Yes | Yes | No | Yes | NA | NA |
| Dolkar et al., 2021^24^ | African | North America | 46 | M | 2 | MCD | Yes | No | No | No | No | Yes | NA |
| Drury et al., 2021^25^ | NA | North America | 55 | M | NA | IgAN-crescentic | NA | Yes | No | No | NA | Yes | NA |
| Ezeakudo, 2022^26^ | African | North America | 63 | F | 14 | FSGS-collapsing glomerulopathy | NA | NA | NA | NA | NA | NA | NA |
| Ferlicot et al., 2021^27^ | NA | Europe | Median 63 (IQR 52-92) | NA | NA | FSGS-collapsing glomerulopathy | NA | NA | NA | NA | NA | NA | NA |
|  | NA | Europe | Median 63 (IQR 52-92) | NA | NA | FSGS-collapsing glomerulopathy | NA | NA | NA | NA | NA | NA | NA |
|  | NA | Europe | Median 63 (IQR 52-92) | NA | NA | FSGS-collapsing glomerulopathy | NA | NA | NA | NA | NA | NA | NA |
|  | NA | Europe | Median 63 (IQR 52-92) | NA | NA | FSGS-collapsing glomerulopathy | NA | NA | NA | NA | NA | NA | NA |
|  | NA | Europe | Median 63 (IQR 52-92) | NA | NA | FSGS-collapsing glomerulopathy | NA | NA | NA | NA | NA | NA | NA |
|  | NA | Europe | Median 63 (IQR 52-92) | NA | NA | FSGS-collapsing glomerulopathy | NA | NA | NA | NA | NA | NA | NA |
|  | NA | Europe | Median 63 (IQR 52-92) | NA | NA | FSGS-collapsing glomerulopathy | NA | NA | NA | NA | NA | NA | NA |
|  | NA | Europe | Median 63 (IQR 52-92) | NA | NA | FSGS-collapsing glomerulopathy | NA | NA | NA | NA | NA | NA | NA |
|  | NA | Europe | Median 63 (IQR 52-92) | NA | NA | FSGS-collapsing glomerulopathy | NA | NA | NA | NA | NA | NA | NA |
|  | NA | Europe | Median 63 (IQR 52-92) | NA | NA | FSGS-collapsing glomerulopathy | NA | NA | NA | NA | NA | NA | NA |
|  | NA | Europe | Median 63 (IQR 52-92) | NA | NA | FSGS-non-collapsing glomerulopathy-Tip variant | NA | NA | NA | NA | NA | NA | NA |
|  | NA | Europe | Median 63 (IQR 52-92) | NA | NA | IgAN | NA | NA | NA | NA | NA | NA | NA |
|  | NA | Europe | Median 63 (IQR 52-92) | NA | NA | MN-PLA2R | NA | NA | NA | NA | NA | NA | NA |
| Fireizen et al., 2021^28^ | NA | North America | 17 | M | 60 | AAV-MPO | NA | No | No | No | NA | Yes | Complete remission |
| Gaillard et al., 2020^29^ | African | North America | 79 | M | 5 | FSGS-collapsing glomerulopathy | NA | No | No | No | NA | Yes | NA |
| Gallagher et al., 2021^30^ | African | North America | 48 | M | NA | FSGS-collapsing glomerulopathy | NA | No | No | No | NA | NA | NA |
| Gambella et al., 2022^31^ | African | Europe | 61 | F | NA | FSGS-collapsing glomerulopathy | Yes | No | No | No | No | NA | NA |
|  | NA | Europe | 89 | F | NA | MCD | Yes | Yes | No | No | No | NA | NA |
|  | NA | Europe | 45 | M | NA | FSGS-collapsing glomerulopathy | NA | Yes | No | No | NA | NA | NA |
|  | NA | Europe | 71 | M | NA | MCD | Yes | Yes | No | No | No | NA | NA |
|  | NA | Europe | 64 | M | NA | MCD | Yes | Yes | No | No | No | NA | NA |
|  | NA | Europe | 15 | M | NA | MCD | Yes | Yes | No | No | No | NA | NA |
| Ganglam et al., 2021^32^ | Asian | North America | 42 | F | NA | LN type IV | Yes | No | No | No | No | Yes | Responding to the treatment |
| Garcia-Vega et al., 2022^33^ | NA | North America | 60 | M | 90 | AAV-MPO | NA | Yes | Yes | No | NA | Yes | Partial remission |
| Giannini et al., 2021^34^ | NA | North America | 71 | F | 30 | PGNMID | NA | No | No | No | NA | Yes | Partial remission |
| Goodman et al., 2021^35^ | African | North America | 53 | F | 17 | FSGS-collapsing glomerulopathy | NA | No | No | No | NA | Yes | NA |
| Guo et al., 2022^36^ | Asian | Asia | 29 | M | 28 | MN-PLA2R | Yes | No | No | No | No | Yes | Partial remission |
| Gupta et al., 2020^37^ | African | North America | 54 | M | NA | FSGS-collapsing glomerulopathy | Yes | Yes | No | No | Yes | Yes | No response |
|  | Asian | North America | 71 | M | 25 | FSGS-collapsing glomerulopathy | Yes | Yes | No | No | Yes | Yes | No response |
| Hale et al., 2020^38^ | African | North America | 56 | M | 7 | FSGS-collapsing glomerulopathy | NA | Yes | No | No | NA | NA | NA |
| Heylen et al., 2021^39^ | NA | Europe | 46 | F | NA | IgAN | NA | No | No | No | NA | NA | NA |
| Hoilat et al., 2021^40^ | African | North America | 63 | F | NA | FSGS-collapsing glomerulopathy | Yes | NA | NA | NA | No | Yes | Responding to the treatment |
| Izci Duran et al., 2021^41^ | NA | Europe | 36 | F | 21 | AAV-PR3 | NA | No | No | No | NA | Yes | NA |
|  | NA | Europe | 26 | M | NA | AAV-pANCA | NA | No | No | No | NA | Yes | NA |
| Izzedine et al., 2020^42^ | African | Europe | 38 | F | 16 | FSGS-collapsing glomerulopathy | NA | NA | NA | NA | NA | NA | NA |
|  | Antilles | Europe | 49 | F | 7 | FSGS-collapsing glomerulopathy | NA | NA | NA | NA | NA | NA | NA |
| Jalalzadeh et al., 2021^43^ | Hispanic/Latino | North America | 46 | F | NA | AAV-pANCA | Yes | Yes | Yes | Ni | No | Yes | NA |
| Jaroszyski et al., 2021^44^ | Caucasian | Europe | 34 | M | NA | AAV-pANCA | NA | Yes | No | No | NA | Yes | Responding to the treatment |
| Jedlowski et al., 2022^45^ | NA | North America | 70 | M | 35 | IgAN-HSP | Yes | No | No | No | No | Yes | Responding to the treatment |
| Kataria et al., 2022^46^ | NA | North America | 79 | F | 21 | AAV- MPO+PR3 | NA | NA | NA | NA | NA | Yes | Responding to the treatment |
| Kawashima et al., 2022^47^ | NA | Asia | 61 | F | 11 | AAV-MPO | NA | Yes | Yes | No | NA | Yes | Responding to the treatment |
| Kazzi et al., 2022^48^ | Hispanic/Latino | North America | 37 | M | 42 | LN type II | Yes | No | No | No | No | Yes | NA |
| Kesiena et al., 2022^49^ | African | North America | 48 | F | 14 | FSGS-collapsing glomerulopathy | Yes | Yes | No | No | No | NA | NA |
| Khan et al., 2021^50^ | NA | North America | 54 | M | NA | C3GN | Yes | No | No | No | No | NA | NA |
| Kissling et al., 2020^51^ | African | North America | 63 | M | 8 | FSGS-collapsing glomerulopathy | Yes | Yes | No | No | Yes | NA | NA |
| Koc et al., 2021^52^ | NA | Asia | 80 | F | 3 | Anti-GBM disease | NA | Yes | Yes | No | NA | Yes | NA |
| Kudose et al., 2021^53^ | African | North America | 54 | F | 13 | FSGS-collapsing glomerulopathy | NA | Yes | Yes | No | NA | Yes | NA |
|  | African | North America | 56 | F | 13 | FSGS-collapsing glomerulopathy | NA | Yes | Yes | No | NA | Yes | No response |
|  | African | North America | 58 | F | 13 | FSGS-collapsing glomerulopathy | NA | Yes | No | No | NA | NA | No response |
|  | African | North America | 37 | F | 14 | FSGS-collapsing glomerulopathy | NA | Yes | Yes | No | NA | NA | No response |
|  | African | North America | 72 | F | 13 | FSGS-collapsing glomerulopathy | NA | Yes | Yes | No | NA | NA | No response |
|  | African | North America | 56 | F | 13 | FSGS-collapsing glomerulopathy | NA | Yes | Yes | No | NA | NA | No response |
|  | African | North America | 59 | F | 90 | FSGS-collapsing glomerulopathy | NA | Yes | Yes | No | NA | NA | No response |
|  | African | North America | 20 | F | 13 | FSGS-non-collapsing glomerulopathy -Tip variant | NA | Yes | No | No | NA | Yes | Partial remission |
|  | African | North America | 59 | F | 21 | MCD | NA | Yes | Yes | No | NA | NA | No response |
|  | African | North America | 54 | M | 13 | Diffused podocytopathy | NA | Yes | Yes | No | NA | NA | NA |
|  | African | North America | 64 | M | 13 | Diffused podocytopathy | NA | Yes | Yes | No | NA | NA | Partial remission |
|  | African | North America | 36 | M | 14 | FSGS | NA | Yes | Yes | No | NA | NA | Partial remission |
|  | African | North America | 35 | M | 21 | FSGS-collapsing glomerulopathy | NA | Yes | Yes | No | NA | Yes | NA |
|  | African | North America | 52 | M | 13 | FSGS-collapsing glomerulopathy | NA | Yes | Yes | No | NA | NA | NA |
|  | African | North America | 69 | M | 13 | FSGS-collapsing glomerulopathy | NA | Yes | Yes | No | NA | NA | NA |
|  | African | North America | 52 | M | 13 | FSGS-collapsing glomerulopathy | NA | Yes | Yes | No | NA | Yes | No response |
|  | African | North America | 64 | M | 120 | FSGS-collapsing glomerulopathy | NA | Yes | Yes | No | NA | NA | No response |
|  | African | North America | 55 | M | 13 | FSGS-collapsing glomerulopathy | NA | Yes | Yes | No | NA | NA | No response |
|  | African | North America | 52 | M | 30 | FSGS-collapsing glomerulopathy | NA | Yes | Yes | No | NA | NA | No response |
|  | African | North America | 61 | M | 30 | FSGS-collapsing glomerulopathy | NA | Yes | Yes | No | NA | NA | No response |
|  | African | North America | 62 | M | 13 | FSGS-collapsing glomerulopathy | NA | Yes | Yes | No | NA | Yes | Partial remission |
|  | African | North America | 68 | M | 13 | FSGS-collapsing glomerulopathy | NA | Yes | Yes | No | NA | Yes | Partial remission |
|  | African | North America | 62 | M | 13 | FSGS-collapsing glomerulopathy | NA | No | Yes | Yes | NA | NA | Partial remission |
|  | African | North America | 54 | M | 13 | FSGS-collapsing glomerulopathy | NA | Yes | No | No | NA | NA | Partial remission |
|  | African | North America | 66 | M | 13 | FSGS-collapsing glomerulopathy | NA | Yes | Yes | No | NA | NA | Partial remission |
|  | African | North America | 57 | M | 28 | FSGS-collapsing glomerulopathy | NA | Yes | Yes | No | NA | NA | Partial remission |
|  | African | North America | 57 | M | 30 | FSGS-collapsing glomerulopathy | NA | Yes | Yes | No | NA | NA | Partial remission |
|  | African | North America | 25 | M | 13 | MCD | NA | Yes | No | No | NA | Yes | Complete remission |
|  | African | North America | 42 | M | 21 | MCD | NA | Yes | No | No | NA | NA | No response |
| Kulkarni et al., 2021^54^ | NA | Asia | 25 | F | 23 | FSGS-collapsing glomerulopathy + IgAN | NA | No | No | Yes | NA | Yes | NA |
| Laboux et al., 2021^55^ | African | Europe | 57 | M | NA | FSGS-collapsing glomerulopathy | NA | Yes | No | No | NA | NA | NA |
| Larsen et al., 2020^56^ | African | South America | 44 | F | 5 | FSGS-collapsing glomerulopathy | Yes | Yes | Yes | No | No | NA | NA |
| Lazoff et al., 2021^57^ | NA | North America | 19 | M | NA | IgAN | NA | NA | NA | NA | NA | Yes | NA |
| Leung et al., 2019^58^ | NA | North America | 19 | M | 150 | Anti-GBM disease | Yes | No | No | No | No | Yes | Partial remission |
| Li et al., 2021^59^ | Caucasian | North America | 30 | M | 42 | IgAN-crescentic | Yes | No | No | No | No | Yes | Complete remission |
| Lind et al., 2021^60^ | NA | North America | 40 | M | 90 | AAV-cANCA | NA | NA | NA | NA | NA | Yes | Responding to the treatment |
| Madanchi et al., 2021^61^ | NA | North America | 53 | M | 120 | AAV -MPO | Yes | Yes | Yes | No | No | Yes | Partial remission |
| Magoon et al., 2020^62^ | African | North America | 28 | F | 7 | FSGS-collapsing glomerulopathy | Yes | Yes | No | No | No | NA | Partial remission |
|  | African | North America | 56 | M | 12 | FSGS-collapsing glomerulopathy | Yes | Yes | No | Yes | No | NA | Partial remission |
| Maldonado et al., 2022^63^ | African | North America | 25 | M | 14 | FSGS-collapsing glomerulopathy | Yes | No | yes | No | No | Yes | NA |
| Malhotra et al., 2020^64^ | African | North America | 64 | M | 11 | FSGS-collapsing glomerulopathy + oxalate nephropathy | Yes | No | Yes | No | No | Yes | NA |
| Malik et al., 2020^65^ | African | North America | 57 | M | 90 | FSGS-collapsing glomerulopathy | Yes | Yes | Yes | No | No | NA | Partial remission |
| Maritati et al., 2021^66^ | NA | Europe | 64 | F | NA | AAV-PR3 | NA | Yes | No | No | NA | Yes | Partial remission |
| May et al., 2021^67^ | NA | Asia, Europe, North America | NA | NA | NA | Anti-GBM disease | NA | NA | NA | NA | NA | NA | NA |
|  | NA | Asia, Europe, North America | NA | NA | NA | Cryoglobulinemic GN | NA | NA | NA | NA | NA | NA | NA |
|  | NA | Asia, Europe, North America | NA | NA | NA | Cryoglobulinemic GN | NA | NA | NA | NA | NA | NA | NA |
|  | NA | Asia, Europe, North America | NA | NA | NA | Cryoglobulinemic GN | NA | NA | NA | NA | NA | NA | NA |
|  | NA | Asia, Europe, North America | NA | NA | NA | Fibrillary GN | NA | NA | NA | NA | NA | NA | NA |
|  | NA | Asia, Europe, North America | NA | NA | NA | FSGS-collapsing glomerulopathy | NA | NA | NA | NA | NA | NA | NA |
|  | NA | Asia, Europe, North America | NA | NA | NA | FSGS-collapsing glomerulopathy | NA | NA | NA | NA | NA | NA | NA |
|  | NA | Asia, Europe, North America | NA | NA | NA | FSGS-collapsing glomerulopathy | NA | NA | NA | NA | NA | NA | NA |
|  | NA | Asia, Europe, North America | NA | NA | NA | FSGS-collapsing glomerulopathy | NA | NA | NA | NA | NA | NA | NA |
|  | NA | Asia, Europe, North America | NA | NA | NA | FSGS-collapsing glomerulopathy | NA | NA | NA | NA | NA | NA | NA |
|  | NA | Asia, Europe, North America | NA | NA | NA | FSGS-collapsing glomerulopathy | NA | NA | NA | NA | NA | NA | NA |
|  | NA | Asia, Europe, North America | NA | NA | NA | FSGS-collapsing glomerulopathy | NA | NA | NA | NA | NA | NA | NA |
|  | NA | Asia, Europe, North America | NA | NA | NA | FSGS-collapsing glomerulopathy | NA | NA | NA | NA | NA | NA | NA |
|  | NA | Asia, Europe, North America | NA | NA | NA | FSGS-collapsing glomerulopathy | NA | NA | NA | NA | NA | NA | NA |
|  | NA | Asia, Europe, North America | NA | NA | NA | FSGS-collapsing glomerulopathy | NA | NA | NA | NA | NA | NA | NA |
|  | NA | Asia, Europe, North America | NA | NA | NA | FSGS-collapsing glomerulopathy | NA | NA | NA | NA | NA | NA | NA |
|  | NA | Asia, Europe, North America | NA | NA | NA | FSGS-collapsing glomerulopathy | NA | NA | NA | NA | NA | NA | NA |
|  | NA | Asia, Europe, North America | NA | NA | NA | FSGS-collapsing glomerulopathy | NA | NA | NA | NA | NA | NA | NA |
|  | NA | Asia, Europe, North America | NA | NA | NA | FSGS-collapsing glomerulopathy | NA | NA | NA | NA | NA | NA | NA |
|  | NA | Asia, Europe, North America | NA | NA | NA | FSGS-collapsing glomerulopathy | NA | NA | NA | NA | NA | NA | NA |
|  | NA | Asia, Europe, North America | NA | NA | NA | FSGS-collapsing glomerulopathy | NA | NA | NA | NA | NA | NA | NA |
|  | NA | Asia, Europe, North America | NA | NA | NA | FSGS-collapsing glomerulopathy | NA | NA | NA | NA | NA | NA | NA |
|  | NA | Asia, Europe, North America | NA | NA | NA | FSGS-collapsing glomerulopathy | NA | NA | NA | NA | NA | NA | NA |
|  | NA | Asia, Europe, North America | NA | NA | NA | FSGS-collapsing glomerulopathy | NA | NA | NA | NA | NA | NA | NA |
|  | NA | Asia, Europe, North America | NA | NA | NA | FSGS-collapsing glomerulopathy | NA | NA | NA | NA | NA | NA | NA |
|  | NA | Asia, Europe, North America | NA | NA | NA | FSGS-collapsing glomerulopathy | NA | NA | NA | NA | NA | NA | NA |
|  | NA | Asia, Europe, North America | NA | NA | NA | FSGS-collapsing glomerulopathy | NA | NA | NA | NA | NA | NA | NA |
|  | NA | Asia, Europe, North America | NA | NA | NA | FSGS-collapsing glomerulopathy | NA | NA | NA | NA | NA | NA | NA |
|  | NA | Asia, Europe, North America | NA | NA | NA | FSGS-collapsing glomerulopathy | NA | NA | NA | NA | NA | NA | NA |
|  | NA | Asia, Europe, North America | NA | NA | NA | FSGS-collapsing glomerulopathy | NA | NA | NA | NA | NA | NA | NA |
|  | NA | Asia, Europe, North America | NA | NA | NA | FSGS-collapsing glomerulopathy | NA | NA | NA | NA | NA | NA | NA |
|  | NA | Asia, Europe, North America | NA | NA | NA | FSGS-collapsing glomerulopathy | NA | NA | NA | NA | NA | NA | NA |
|  | NA | Asia, Europe, North America | NA | NA | NA | FSGS-collapsing glomerulopathy | NA | NA | NA | NA | NA | NA | NA |
|  | NA | Asia, Europe, North America | NA | NA | NA | FSGS-collapsing glomerulopathy | NA | NA | NA | NA | NA | NA | NA |
|  | NA | Asia, Europe, North America | NA | NA | NA | FSGS-collapsing glomerulopathy | NA | NA | NA | NA | NA | NA | NA |
|  | NA | Asia, Europe, North America | NA | NA | NA | FSGS-collapsing glomerulopathy | NA | NA | NA | NA | NA | NA | NA |
|  | NA | Asia, Europe, North America | NA | NA | NA | FSGS-collapsing glomerulopathy | NA | NA | NA | NA | NA | NA | NA |
|  | NA | Asia, Europe, North America | NA | NA | NA | FSGS-collapsing glomerulopathy | NA | NA | NA | NA | NA | NA | NA |
|  | NA | Asia, Europe, North America | NA | NA | NA | FSGS-collapsing glomerulopathy | NA | NA | NA | NA | NA | NA | NA |
|  | NA | Asia, Europe, North America | NA | NA | NA | FSGS-collapsing glomerulopathy | NA | NA | NA | NA | NA | NA | NA |
|  | NA | Asia, Europe, North America | NA | NA | NA | FSGS-collapsing glomerulopathy | NA | NA | NA | NA | NA | NA | NA |
|  | NA | Asia, Europe, North America | NA | NA | NA | FSGS-collapsing glomerulopathy | NA | NA | NA | NA | NA | NA | NA |
|  | NA | Asia, Europe, North America | NA | NA | NA | FSGS-collapsing glomerulopathy | NA | NA | NA | NA | NA | NA | NA |
|  | NA | Asia, Europe, North America | NA | NA | NA | FSGS-collapsing glomerulopathy | NA | NA | NA | NA | NA | NA | NA |
|  | NA | Asia, Europe, North America | NA | NA | NA | FSGS-collapsing glomerulopathy | NA | NA | NA | NA | NA | NA | NA |
|  | NA | Asia, Europe, North America | NA | NA | NA | FSGS-collapsing glomerulopathy | NA | NA | NA | NA | NA | NA | NA |
|  | NA | Asia, Europe, North America | NA | NA | NA | FSGS-collapsing glomerulopathy | NA | NA | NA | NA | NA | NA | NA |
|  | NA | Asia, Europe, North America | NA | NA | NA | FSGS-collapsing glomerulopathy | NA | NA | NA | NA | NA | NA | NA |
|  | NA | Asia, Europe, North America | NA | NA | NA | FSGS-collapsing glomerulopathy | NA | NA | NA | NA | NA | NA | NA |
|  | NA | Asia, Europe, North America | NA | NA | NA | FSGS-collapsing glomerulopathy | NA | NA | NA | NA | NA | NA | NA |
|  | NA | Asia, Europe, North America | NA | NA | NA | FSGS-collapsing glomerulopathy | NA | NA | NA | NA | NA | NA | NA |
|  | NA | Asia, Europe, North America | NA | NA | NA | FSGS-collapsing glomerulopathy | NA | NA | NA | NA | NA | NA | NA |
|  | NA | Asia, Europe, North America | NA | NA | NA | FSGS-collapsing glomerulopathy | NA | NA | NA | NA | NA | NA | NA |
|  | NA | Asia, Europe, North America | NA | NA | NA | FSGS-collapsing glomerulopathy | NA | NA | NA | NA | NA | NA | NA |
|  | NA | Asia, Europe, North America | NA | NA | NA | FSGS-collapsing glomerulopathy | NA | NA | NA | NA | NA | NA | NA |
|  | NA | Asia, Europe, North America | NA | NA | NA | FSGS-collapsing glomerulopathy | NA | NA | NA | NA | NA | NA | NA |
|  | NA | Asia, Europe, North America | NA | NA | NA | FSGS-collapsing glomerulopathy | NA | NA | NA | NA | NA | NA | NA |
|  | NA | Asia, Europe, North America | NA | NA | NA | FSGS-collapsing glomerulopathy | NA | NA | NA | NA | NA | NA | NA |
|  | NA | Asia, Europe, North America | NA | NA | NA | FSGS-collapsing glomerulopathy | NA | NA | NA | NA | NA | NA | NA |
|  | NA | Asia, Europe, North America | NA | NA | NA | FSGS-collapsing glomerulopathy | NA | NA | NA | NA | NA | NA | NA |
|  | NA | Asia, Europe, North America | NA | NA | NA | FSGS-collapsing glomerulopathy | NA | NA | NA | NA | NA | NA | NA |
|  | NA | Asia, Europe, North America | NA | NA | NA | FSGS-collapsing glomerulopathy | NA | NA | NA | NA | NA | NA | NA |
|  | NA | Asia, Europe, North America | NA | NA | NA | FSGS-collapsing glomerulopathy | NA | NA | NA | NA | NA | NA | NA |
|  | NA | Asia, Europe, North America | NA | NA | NA | FSGS-collapsing glomerulopathy | NA | NA | NA | NA | NA | NA | NA |
|  | NA | Asia, Europe, North America | NA | NA | NA | FSGS-collapsing glomerulopathy | NA | NA | NA | NA | NA | NA | NA |
|  | NA | Asia, Europe, North America | NA | NA | NA | FSGS-collapsing glomerulopathy | NA | NA | NA | NA | NA | NA | NA |
|  | NA | Asia, Europe, North America | NA | NA | NA | FSGS-collapsing glomerulopathy | NA | NA | NA | NA | NA | NA | NA |
|  | NA | Asia, Europe, North America | NA | NA | NA | FSGS-collapsing glomerulopathy | NA | NA | NA | NA | NA | NA | NA |
|  | NA | Asia, Europe, North America | NA | NA | NA | FSGS-collapsing glomerulopathy | NA | NA | NA | NA | NA | NA | NA |
|  | NA | Asia, Europe, North America | NA | NA | NA | FSGS-collapsing glomerulopathy | NA | NA | NA | NA | NA | NA | NA |
|  | NA | Asia, Europe, North America | NA | NA | NA | FSGS-collapsing glomerulopathy | NA | NA | NA | NA | NA | NA | NA |
|  | NA | Asia, Europe, North America | NA | NA | NA | FSGS-collapsing glomerulopathy | NA | NA | NA | NA | NA | NA | NA |
|  | NA | Asia, Europe, North America | NA | NA | NA | FSGS-collapsing glomerulopathy | NA | NA | NA | NA | NA | NA | NA |
|  | NA | Asia, Europe, North America | NA | NA | NA | FSGS-collapsing glomerulopathy | NA | NA | NA | NA | NA | NA | NA |
|  | NA | Asia, Europe, North America | NA | NA | NA | FSGS-collapsing glomerulopathy | NA | NA | NA | NA | NA | NA | NA |
|  | NA | Asia, Europe, North America | NA | NA | NA | FSGS-non-collapsing glomerulopathy | NA | NA | NA | NA | NA | NA | NA |
|  | NA | Asia, Europe, North America | NA | NA | NA | IgAN | NA | NA | NA | NA | NA | NA | NA |
|  | NA | Asia, Europe, North America | NA | NA | NA | IgAN | NA | NA | NA | NA | NA | NA | NA |
|  | NA | Asia, Europe, North America | NA | NA | NA | IgAN | NA | NA | NA | NA | NA | NA | NA |
|  | NA | Asia, Europe, North America | NA | NA | NA | IgAN | NA | NA | NA | NA | NA | NA | NA |
|  | NA | Asia, Europe, North America | NA | NA | NA | IgAN | NA | NA | NA | NA | NA | NA | NA |
|  | NA | Asia, Europe, North America | NA | NA | NA | IgAN | NA | NA | NA | NA | NA | NA | NA |
|  | NA | Asia, Europe, North America | NA | NA | NA | IgAN | NA | NA | NA | NA | NA | NA | NA |
|  | NA | Asia, Europe, North America | NA | NA | NA | LN type unknown | NA | NA | NA | NA | NA | NA | NA |
|  | NA | Asia, Europe, North America | NA | NA | NA | LN type unknown | NA | NA | NA | NA | NA | NA | NA |
|  | NA | Asia, Europe, North America | NA | NA | NA | LN type unknown | NA | NA | NA | NA | NA | NA | NA |
|  | NA | Asia, Europe, North America | NA | NA | NA | LN type unknown | NA | NA | NA | NA | NA | NA | NA |
|  | NA | Asia, Europe, North America | NA | NA | NA | LN type unknown | NA | NA | NA | NA | NA | NA | NA |
|  | NA | Asia, Europe, North America | NA | NA | NA | LN type unknown | NA | NA | NA | NA | NA | NA | NA |
|  | NA | Asia, Europe, North America | NA | NA | NA | MCD | NA | NA | NA | NA | NA | NA | NA |
|  | NA | Asia, Europe, North America | NA | NA | NA | MCD | NA | NA | NA | NA | NA | NA | NA |
|  | NA | Asia, Europe, North America | NA | NA | NA | MCD | NA | NA | NA | NA | NA | NA | NA |
|  | NA | Asia, Europe, North America | NA | NA | NA | MCD | NA | NA | NA | NA | NA | NA | NA |
|  | NA | Asia, Europe, North America | NA | NA | NA | MCD | NA | NA | NA | NA | NA | NA | NA |
|  | NA | Asia, Europe, North America | NA | NA | NA | MCD | NA | NA | NA | NA | NA | NA | NA |
|  | NA | Asia, Europe, North America | NA | NA | NA | MCD | NA | NA | NA | NA | NA | NA | NA |
|  | NA | Asia, Europe, North America | NA | NA | NA | MCD | NA | NA | NA | NA | NA | NA | NA |
|  | NA | Asia, Europe, North America | NA | NA | NA | MCD | NA | NA | NA | NA | NA | NA | NA |
|  | NA | Asia, Europe, North America | NA | NA | NA | MCD | NA | NA | NA | NA | NA | NA | NA |
|  | NA | Asia, Europe, North America | NA | NA | NA | MCD | NA | NA | NA | NA | NA | NA | NA |
|  | NA | Asia, Europe, North America | NA | NA | NA | MCD | NA | NA | NA | NA | NA | NA | NA |
|  | NA | Asia, Europe, North America | NA | NA | NA | Membranous like nephropathy with monoclonal IgG Kappa deposits (MGMID) | NA | NA | NA | NA | NA | NA | NA |
|  | NA | Asia, Europe, North America | NA | NA | NA | MN - Antigen type unknown | NA | NA | NA | NA | NA | NA | NA |
|  | NA | Asia, Europe, North America | NA | NA | NA | MN - Antigen type unknown | NA | NA | NA | NA | NA | NA | NA |
|  | NA | Asia, Europe, North America | NA | NA | NA | MN - Antigen type unknown | NA | NA | NA | NA | NA | NA | NA |
|  | NA | Asia, Europe, North America | NA | NA | NA | MN - Antigen type unknown | NA | NA | NA | NA | NA | NA | NA |
|  | NA | Asia, Europe, North America | NA | NA | NA | MN - Antigen type unknown | NA | NA | NA | NA | NA | NA | NA |
|  | NA | Asia, Europe, North America | NA | NA | NA | MN - Antigen type unknown | NA | NA | NA | NA | NA | NA | NA |
|  | NA | Asia, Europe, North America | NA | NA | NA | MN - Antigen type unknown | NA | NA | NA | NA | NA | NA | NA |
|  | NA | Asia, Europe, North America | NA | NA | NA | MN - Antigen type unknown | NA | NA | NA | NA | NA | NA | NA |
|  | NA | Asia, Europe, North America | NA | NA | NA | MN - Antigen type unknown | NA | NA | NA | NA | NA | NA | NA |
|  | NA | Asia, Europe, North America | NA | NA | NA | MN - Antigen type unknown | NA | NA | NA | NA | NA | NA | NA |
|  | NA | Asia, Europe, North America | NA | NA | NA | MN - Antigen type unknown | NA | NA | NA | NA | NA | NA | NA |
|  | NA | Asia, Europe, North America | NA | NA | NA | MN - Antigen type unknown | NA | NA | NA | NA | NA | NA | NA |
|  | NA | Asia, Europe, North America | NA | NA | NA | Pauci-immune GN- unknown cause | NA | NA | NA | NA | NA | NA | NA |
|  | NA | Asia, Europe, North America | NA | NA | NA | Pauci-immune GN- unknown cause | NA | NA | NA | NA | NA | NA | NA |
|  | NA | Asia, Europe, North America | NA | NA | NA | Pauci-immune GN- unknown cause | NA | NA | NA | NA | NA | NA | NA |
|  | NA | Asia, Europe, North America | NA | NA | NA | Pauci-immune GN- unknown cause | NA | NA | NA | NA | NA | NA | NA |
|  | NA | Asia, Europe, North America | NA | NA | NA | Pauci-immune GN- unknown cause | NA | NA | NA | NA | NA | NA | NA |
|  | NA | Asia, Europe, North America | NA | NA | NA | Pauci-immune GN- unknown cause | NA | NA | NA | NA | NA | NA | NA |
|  | NA | Asia, Europe, North America | NA | NA | NA | Pauci-immune GN- unknown cause | NA | NA | NA | NA | NA | NA | NA |
|  | NA | Asia, Europe, North America | NA | NA | NA | Pauci-immune GN- unknown cause | NA | NA | NA | NA | NA | NA | NA |
|  | NA | Asia, Europe, North America | NA | NA | NA | Pauci-immune GN- unknown cause | NA | NA | NA | NA | NA | NA | NA |
|  | NA | Asia, Europe, North America | NA | NA | NA | Pauci-immune GN- unknown cause | NA | NA | NA | NA | NA | NA | NA |
|  | NA | Asia, Europe, North America | NA | NA | NA | Pauci-immune GN- unknown cause | NA | NA | NA | NA | NA | NA | NA |
|  | NA | Asia, Europe, North America | NA | NA | NA | Pauci-immune GN- unknown cause | NA | NA | NA | NA | NA | NA | NA |
|  | NA | Asia, Europe, North America | NA | NA | NA | PGNMID | NA | NA | NA | NA | NA | NA | NA |
|  | NA | Asia, Europe, North America | NA | NA | NA | PGNMID | NA | NA | NA | NA | NA | NA | NA |
|  | NA | Asia, Europe, North America | NA | NA | NA | PGNMID | NA | NA | NA | NA | NA | NA | NA |
|  | NA | Asia, Europe, North America | NA | NA | NA | PGNMID | NA | NA | NA | NA | NA | NA | NA |
|  | NA | Asia, Europe, North America | NA | NA | NA | PGNMID | NA | NA | NA | NA | NA | NA | NA |
| Mbengue et al., 2022^68^ | African | Africa | 70 | F | 14 | AAV-MPO | Yes | NA | NA | NA | No | Yes | No response |
| Meliambro et al., 2021^69^ | African | North America | 50 | F | 35 | FSGS-collapsing glomerulopathy | Yes | Yes | No | No | No | NA | NA |
| Miao et al., 2021^70^ | Hispanic/Latino | North America | 81 | M | 10 | MN - Antigen type unknown | Yes | Yes | Yes | No | No | No | NA |
| Moeinzadeh et al., 2020^71^ | NA | Asia | 25 | M | 6 | AAV-cANCA | NA | NA | NA | NA | NA | Yes | Partial remission |
| Naqvi et al., 2021^72^ | NA | North America | Median 55 | F | NA | Cryoglobulinemic GN - type 1 | NA | NA | NA | NA | NA | Yes |  |
| Nasar et al., 2021^73^ | African | North America | Median 55 | M | NA | FSGS-collapsing glomerulopathy | Yes | Yes | Yes | No | No | NA | Complete remission |
|  | African | North America | Median 55 | M | NA | FSGS-collapsing glomerulopathy | Yes | Yes | Yes | No | No | NA | Complete remission |
|  | African | North America | Median 55 | M | NA | FSGS-collapsing glomerulopathy | Yes | Yes | Yes | No | No | NA | No response |
|  | African | North America | Median 55 | M | NA | FSGS-collapsing glomerulopathy | Yes | Yes | Yes | No | No | NA | No response |
|  | African | North America | Median 55 | M | NA | FSGS-collapsing glomerulopathy | NA | Yes | Yes | No | NA | NA | No response |
|  | African | North America | Median 55 | M | NA | FSGS-collapsing glomerulopathy | Yes | Yes | Yes | No | No | NA | Partial remission |
|  | African | North America | Median 55 | M | NA | FSGS-collapsing glomerulopathy | Yes | Yes | Yes | No | No | NA | Partial remission |
|  | African | #REF! | NA | M | NA | FSGS-collapsing glomerulopathy +MN-PLA2R negative | Yes | Yes | Yes | No | No | NA | No response |
|  | Caucasian | North America | NA | M | NA | IgAN - HSP nephritis-crescentic | Yes | Yes | Yes | No | No | NA | NA |
|  | Hispanic/Latino | North America | NA | M | NA | MN- PLA2R negative | Yes | Yes | Yes | No | No | NA | NA |
| Nlandu et al., 2020^74^ | African | Africa | 48 | M | 49 | FSGS-collapsing glomerulopathy | NA | Yes | No | No | NA | Yes | Partial remission |
| Noble et al., 2020^75^ | African | North America | 54 | M | 19 | FSGS-collapsing glomerulopathy | Yes | Yes | No | No | No | NA | Partial remission |
| Nomura et al., 2022^76^ | Hispanic/Latino | North America | 18 | F | NA | MCD | Yes | NA | NA | NA | No | Yes | No response |
| Pasilan et al., 2022^77^ | Asian | Asia | 40 | F | NA | MCD | Yes | No | No | No | No | Yes | Complete remission |
|  | Asian | Asia | 61 | M | 120 | MCD | Yes | No | Yes | No | No | Yes | Complete remission |
| Peleg et al., 2020^78^ | African | North America | 46 | M | 30 | FSGS-collapsing glomerulopathy | NA | Yes | Yes | No | NA | Yes | NA |
| Peracha et al., 2022^79^ | NA | Europe | Median 60 | 4F, 3M | NA | FSGS-collapsing glomerulopathy | NA | NA | NA | NA | NA | NA | No response |
|  | NA | Europe | Median 60 | 4F, 3M | NA | FSGS-collapsing glomerulopathy | NA | NA | NA | NA | NA | NA | Partial remission |
|  | NA | Europe | Median 60 | 4F, 3M | NA | FSGS-collapsing glomerulopathy | NA | NA | NA | NA | NA | NA | Partial remission |
|  | NA | Europe | Median 60 | 4F, 3M | NA | FSGS-collapsing glomerulopathy | NA | NA | NA | NA | NA | NA | Partial remission |
|  | NA | Europe | Median 60 | 4F, 3M | NA | FSGS-collapsing glomerulopathy | NA | NA | NA | NA | NA | NA | Partial remission |
|  | NA | Europe | Median 60 | 4F, 3M | NA | FSGS-collapsing glomerulopathy | NA | NA | NA | NA | NA | NA | Partial remission |
|  | NA | Europe | Median 65 | 4F, 3M | NA | FSGS-collapsing glomerulopathy | NA | NA | NA | NA | NA | NA | Partial remission |
| Perez et al., 2021^80^ | NA | Europe | 88 | M | 56 | IgAN | Yes | No | Yes | Yes | Yes | Yes | Complete remission |
| Román et al., 2021^81^ | NA | Europe | 56 | M | 14 | FSGS - non-collapsing glomerulopathy -NOS variant | NA | NA | NA | NA | NA | Yes | Responding to the treatment |
| Rosaly et al., 2021^82^ | NA | North America | 65 | F | NA | AAV – pANCA | NA | No | No | No | NA | Yes | Responding to the treatment |
| Rossi et al., 2020^83^ | NA | Europe | 49 | M | NA | FSGS-collapsing glomerulopathy | Yes | Yes | Yes | No | No | Yes | Responding to the treatment |
| Roy et al., 2021^84^ | African | North America | 65 | M | NA | FSGS-collapsing glomerulopathy | Yes | NA | NA | NA | No | Yes | Complete remission |
| Sandhu et al., 2021^85^ | NA | Asia | 22 | M | NA | IgAN | NA | No | No | No | NA | Yes | Responding to the treatment |
| Sebastian et al., 2021^86^ | NA | Asia | 36 | F | 28 | Anti-GBM disease | NA | No | No | No | NA | Yes | NA |
|  | NA | Asia | 32 | F | 28 | Anti-GBM disease | NA | No | No | No | NA | Yes | Responding to the treatment |
|  | NA | Asia | 18 | M | 56 | Anti-GBM disease | NA | No | No | No | NA | Yes | NA |
|  | NA | Asia | 52 | M | 42 | Anti-GBM disease | NA | No | No | No | NA | Yes | No response |
| Selvaraj et al., 2021^87^ | NA | North America | 60 | F | 35 | AAV - PR3 | NA | Yes | Yes | No | NA | Yes | Responding to the treatment |
| Seshan et al., 2020^88^ | Asian1, African 9, Hispanic 1 | North America | 43(25-73) | 5F, 5M | NA | FSGS-collapsing glomerulopathy | Yes | No | Yes | No | Yes | NA | NA |
|  | Asian1, African 9, Hispanic 1 | North America | 43(25-73) | 5F, 5M | NA | FSGS-collapsing glomerulopathy | Yes | No | Yes | No | Yes | NA | NA |
|  | Asian1, African 9, Hispanic 1 | North America | 43(25-73) | 5F, 5M | NA | FSGS-collapsing glomerulopathy | Yes | No | Yes | No | Yes | NA | NA |
|  | Asian1, African 9, Hispanic 1 | North America | 43(25-73) | 5F, 5M | NA | FSGS-collapsing glomerulopathy | Yes | No | Yes | No | Yes | NA | NA |
|  | Asian1, African 9, Hispanic 1 | North America | 43(25-73) | 5F, 5M | NA | FSGS-collapsing glomerulopathy | Yes | No | Yes | No | Yes | NA | NA |
|  | Asian1, African 9, Hispanic 1 | North America | 43(25-73) | 5F, 5M | NA | FSGS-collapsing glomerulopathy | Yes | No | Yes | No | Yes | NA | NA |
|  | Asian1, African 9, Hispanic 1 | North America | 43(25-73) | 5F, 5M | NA | FSGS-collapsing glomerulopathy | Yes | No | Yes | No | Yes | NA | NA |
|  | Asian1, African 9, Hispanic 1 | North America | 43(25-73) | 5F, 5M | NA | FSGS-collapsing glomerulopathy | Yes | No | Yes | No | Yes | NA | NA |
|  | Asian1, African 9, Hispanic 1 | North America | 43(25-73) | 5F, 5M | NA | FSGS-collapsing glomerulopathy | Yes | No | Yes | No | Yes | NA | NA |
|  | Asian1, African 9, Hispanic 1 | North America | 43(25-73) | 5F, 5M | NA | FSGS-collapsing glomerulopathy | Yes | No | Yes | No | Yes | NA | NA |
| Sethi et al., 2021^89^ | Caucasian | North America | 25 | F | 35 | MPGN | Yes | Yes | Yes | Yes | No | Yes | NA |
| Sharma et al., 2020a^90^ | African | North America | 49 | M | NA | FSGS-collapsing glomerulopathy | NA | No | No | No | NA | NA | NA |
|  | African | North America | 67 | F | NA | FSGS-collapsing glomerulopathy | NA | NA | NA | NA | NA | NA | NA |
| Sharma et al., 2020b^91^ | African | North America | 77 | F | 21 | FSGS-collapsing glomerulopathy | Yes | Yes | No | No | No | NA | Complete remission |
|  | African | North America | 64 | M | NA | AAV -MPO | NA | Yes | No | No | NA | Yes | Complete remission |
| Sherchan et al., 2020^92^ | African | North America | 25 | F | 27 | FSGS-collapsing glomerulopathy | NA | Yes | No | No | NA | NA | NA |
|  | African | North America | 42 | F | NA | FSGS-collapsing glomerulopathy | Yes | Yes | No | No | Yes | NA | NA |
| Sherchan et al., 2021^93^ | African | North America | 25 | F | 25 | FSGS-collapsing glomerulopathy | Yes | Yes | No | No | Yes | Yes | Responding to the treatment |
| Shetty et al., 2021^94^ | African | North America | 60 (48-64) | 2F, 3M | NA | FSGS-collapsing glomerulopathy | Yes | NA | NA | NA | No | Yes | Partial/Complete remission |
|  | African | North America | 57 (48-64) | 2F, 3M | NA | FSGS-collapsing glomerulopathy | Yes | NA | NA | NA | No | Yes | Partial/Complete remission |
|  | African | North America | 58 (48-64) | 2F, 3M | NA | FSGS-collapsing glomerulopathy | Yes | NA | NA | NA | No | Yes | Partial/Complete remission |
|  | African | North America | 59 (48-64) | 2F, 3M | NA | FSGS-collapsing glomerulopathy | Yes | NA | NA | NA | No | Yes | Partial/Complete remission |
|  | African | North America | 61 (48-64) | 2F, 3M | NA | FSGS-collapsing glomerulopathy | Yes | NA | NA | NA | No | No | Partial/Complete remission |
| Shieh et al., 2022^95^ | NA | North America | 71 | F | NA | PGNMID with monoclonal IgG3-kappa deposits | NA | NA | NA | NA | NA | Yes | Responding to the treatment |
| Spinella et al., 2021^96^ | African | North America | 57 | F | NA | FSGS-Collapsing glomerulopathy | Yes | NA | NA | NA | No | Yes | NA |
| Suso et al., 2020^97^ | NA | Europe | 78 | M | NA | IgAN | Yes | No | No | No | No | Yes | Responding to the treatment |
| Tancredi et al., 2021^98^ | African | North America | 38 | F | NA | FSGS-collapsing glomerulopathy | NA | NA | NA | NA | NA | NA | NA |
| Thilakaratne et al., 2022^99^ | Caucasian | North America | 30 | M | 180 | FSGS - non-collapsing glomerulopathy -NOS variant | Yes | NA | NA | NA | No | Yes | Responding to the treatment |
| Uppal et al., 2020^100^ | African | North America | 64 | M | NA | AAV-MPO | Yes | Yes | No | No | No | Yes | Responding to the treatment |
|  | Asian | North America | 46 | M | NA | AAV-PR3 | Yes | Yes | Yes | No | No | Yes | Complete remission |
| Vivekanand et al., 2022^101^ | Asian | Asia | 16 | M | 90 | IgAN- crescentic | NA | Yes | No | No | NA | Yes | No response |
| Waseem et al., 2021^102^ | NA | North America | 53 | F | 90 | Pauci-immune GN - unknown cause | NA | NA | NA | NA | NA | Yes | Responding to the treatment |
| Wu et al., 2020^103^ | African | North America | 65 | F | 12 | FSGS-Collapsing glomerulopathy | Yes | Yes | Yes | No | No | NA | NA |
|  | African | North America | 64 | F | 7 | FSGS-Collapsing glomerulopathy | Yes | Yes | No | No | No | NA | Partial remission |
|  | African | North America | 37 | M | 14 | FSGS-Collapsing glomerulopathy | Yes | Yes | No | No | No | NA | NA |
|  | African | North America | 63 | M | 14 | FSGS-Collapsing glomerulopathy | Yes | Yes | No | No | No | NA | No response |
|  | African | North America | 44 | M | 11 | FSGS-Collapsing glomerulopathy | Yes | Yes | No | No | No | NA | No response |
|  | African | North America | 56 | M | 6 | FSGS-Collapsing glomerulopathy | NA | Yes | No | No | NA | NA | Partial remission |
| Yonishi et al., 2022^104^ | NA | Asia | 74 | M | 180 | IgAN | NA | No | No | No | NA | Yes | Complete remission |
| Zakrocka et al., 2022^105^ | NA | Europe | 59 | M | 10 | AAV-MPO | NA | Yes | No | No | NA | Yes | Complete remission |
| Zemke et al., 2020^106^ | African | North America | 51 | F | 13 | FSGS-Collapsing glomerulopathy | NA | Yes | No | No | NA | Yes | Responding to the treatment |

COVID-19 = Coronavirus Disease 2019, M=Male, F=Female, GD = Glomerular Disease, EM = Electron microscopy, ATN = Acute tubular necrosis, ATIN = Acute tubulointerstitial nephritis, TMA = Thrombotic microangiopathy, FSGS = Focal Segmental Glomerulosclerosis, MCD = Minimal Change Disease, IgAN = Immunoglobulin A Nephropathy, HSP = Henoch, Schoenlein purpura, HUS = Haemolytic uremic syndrome, MPGN = Membranoproliferative Glomerulonephritis, LN = Lupus Nephritis, Anti-GBM = Anti glomerular membrane, GN = Glomerulonephritis, AAV = Anti-neutrophil cytoplasmic antibodies Associated Vasculitis, MPO = myeloperoxidase, PR3 = Proteinase 3, C3GN = Complement 3 Glomerulonephritis, PGNMID = Proliferative GN with Monoclonal Immunoglobulin Deposition, NA = not available.

**References**

1. Adapa SR, Pokal M, Sangani V, Roy S. Collapsing glomerulopathy in COVID patient-A case report. ASAIO J. 2021; 67: 69.

2. Afonso RS, Calças Marques R, Borges H, Carias E, Domingos AT, Cabrita A, Sampaio S, Silva AP. Tip lesion variant of focal and segmental glomerulosclerosis in a COVID-19 patient. Case Rep Nephrol Dial. 2022; 12: 248-254.

3. Akilesh S, Nast CC, Yamashita M, Henriksen K, Charu V, Troxell ML, Kambham N, Bracamonte E, Houghton D, Ahmed NI, Chong CC, Thajudeen B, Rehman S, Khoury F, Zuckerman JE, Gitomer J, Raguram PC, Mujeeb S, Schwarze U, Shannon MB, De Castro I, Alpers CE, Najafian B, Nicosia RF, Andeen NK, Smith KD. Multicenter clinicopathologic correlation of kidney biopsies performed in COVID-19 patients presenting with acute kidney injury or proteinuria. Am J Kidney Dis. 2021;77: 82-93.

4. Akrawi S, Sabbouh T, Janom K, Rao PS, Lapedis CJ. Catastrophic COVID-19-associated nephropathy (COVAN) in an asymptomatic patient. Kidney week 2021 - poster. J Am Soc Nephrol. 2021; 32: 448.

5. Alawad MJ, Subahi EA, Al-Ani HA, Taha NM, Kamal I. A case of crescentic glomerulonephritis in a patient with COVID-19 infection: A case report and literature review. Medicine (Baltimore). 2022; 101: e28754.

6. Allena N, Patel J, Nader G, Patel M, Medvedovsky B. A rare case of SARS-CoV-2-induced microscopic polyangiitis. Cureus. 2021; 13: e15259.

7. Alotaibi M, Ellis C, Wadhwani S, Peleg Y. A rare case of granulomatous interstitial nephritis in a patient with COVID-19. J Investig Med High Impact Case Rep. 2022; 10: 23247096221114517.

8. Amin S, Rahim F, Noor M, Wahab A, Qureshi SA. COVID-19 associated illnesses from alveoli to glomeruli: A case report. Cureus. 2022; 14: e25670.

9. Apaydın H, Güven SC, Doğan I, Çolak A, Erten Ş. ANCA-positive IgA nephropathy presented as alveolar hemorrhage in a COVID-19 patient. Annals of Clinical and Analytical Medicine. 2021; 236-240.

10. Asma H, Mariem BS, Nouha BM, Manel BS, Insaf H, Ahmed L, Mouna H, Sabra A, Habib S. pos-930 - Anca associated vasculitis with crescentic glomerulonephritis complicating covid 19 in a 72-year-old man. Kidney International Reports. 2022; 7: S406.

11. Basiratnia M, Derakhshan D, Yeganeh BS, Derakhshan A. Acute necrotizing glomerulonephritis associated with COVID-19 infection: report of two pediatric cases. Pediatr Nephrol. 2021; 36: 1019-1023.

12. Borrero-Arvelo A, Sebastian LM, Collazo-Maldonado RL. Cause or not: IGA-dominant infectious-related glomerulonephritis in a patient infected with COVID-19. J Am Soc Nephrol. 2021; 91.

13. Boudhabhay I, Rabant M, Roumenina LT, Coupry LM, Poillerat V, Marchal A, Frémeaux-Bacchi V, El Karoui K, Monchi M, Pourcine F. Case Report: Adult post-COVID-19 multisystem inflammatory syndrome and thrombotic microangiopathy. Front Immunol. 2021; 12: 680567.

14. Üner ME, Ayhan EM. Kidney biopsy findings in SARS-Cov2 infected patients: a single center case series of post/co-Covid glomerulopathies & kidney injury.

15. Chandler Z, Patel N, Kahn C, Hasan I, Jaikaransingh V. Microscopic polyangiitis with pulmonary-renal syndrome following sars-cov-2 infection. Am J Kidney Dis. 2021; 583.

16. Chang MA, Babayev R. The tale of two collapsing glomerulopathies associated with COVID-19 in stamford hospital. J Am Soc Nephrol 2021; 776.

17. Chargui S, Mbarek M, Harzallah A, Aouidia R, Ouinissi M, Abdallah TB. POS-172 - Renal thrombotic microangiopathy, ANCA vasculitis and COVID 19 infection: an intriguing association. Kidney International Reports. 2021; 6: S70.

18. Christodoulou M, Iatridi F, Chalkidis G, Lioulios G, Nikolaidou C, Badis K, Fylaktou A, Papagianni A, Stangou M. ANCA-associated vasculitis may result as a complication to both SARS-CoV-2 infection and vaccination. Life (Basel). 2022; 12: 1072.

19. D'Ambrosio D, Cesaro A, Del Prete I, Vatiero V, Damiano S, Ievoli F. Rapidly progressive paucimmune glomerulonephritis following COVID-19 infection. Italian Journal of Medicine. 2022; 5.

20. Danis R, Gunay E, Yuksel E, Kaya S, Kılıc J, Kacar E, Senol A, Deniz Altıntas D, Yıldırım MS. Successful treatment of covid-19-related immune- complex glomerulonephritis, case report. Iran J Kidney Dis. 2022;16: 147-151.

21. Hoilat GJ, Das G, Shahnawaz M, Shanley P, Bukhari SH. COVID-19 induced collapsing glomerulopathy and role of APOL1. QJM: An International Journal of Medicine. 2021; 114: 263-264.

22. De Fabritiis M, Angelini ML, Fabbrizio B, Cenacchi G, Americo C, Cristino S, Lifrieri MF, Cappuccilli M, Spazzoli A, Zambianchi L, Mosconi G. Renal thrombotic microangiopathy in concurrent COVID-19 vaccination and infection. Pathogens. 2021; 10: 1045.

23. Deshmukh S, Zhou XJ, Hiser W. Collapsing glomerulopathy in a patient of Indian descent in the setting of COVID-19. Ren Fail. 2020; 42: 877-880.

24. Dolkar T, Shrivastava D, Athreya A, Nway N. COVID-19 associated minimal change disease nephrotic syndrome. Am J Kidney Dis. 2021; 589.

25. Drury Z, Al-Rabadi L, Gregory MC, Gilligan S, Ohri R, Penafiel MP, Abraham J. Iga nephropathy post COVID-19 infection. J Am Soc Nephrol. 2021; 769-770.

26. Ezeakudo N, Lee P, Patel N. Sudden onset covid-associated nephropathy in a vaccinated patient. Am J Kidney Dis. 2022; Vol. 79, No. 4, S92-93.

27. Ferlicot S, Jamme M, Gaillard F, Oniszczuk J, Couturier A, May O, Grünenwald A, Sannier A, Moktefi A, Le Monnier O, Petit-Hoang C, Maroun N, Brodin-Sartorius A, Michon A, Dobosziewicz H, Andreelli F, Guillet M, Izzedine H, Richard C, Dekeyser M, Arrestier R, Sthelé T, Lefèvre E, Mathian A, Legendre C, Mussini C, Verpont MC, Pallet N, Amoura Z, Essig M, Snanoudj R, Brocheriou-Spelle I, François H, Belenfant X, Geri G, Daugas E, Audard V, Buob D, Massy ZA, Zaidan M; AP-HP/Universities/Inserm COVID-19 research collaboration. The spectrum of kidney biopsies in hospitalized patients with COVID-19, acute kidney injury, and/or proteinuria. Nephrol Dial Transplant. 2021; gfab 042.

28. Fireizen Y, Shahriary C, Imperial ME, Randhawa I, Nianiaris N, Ovunc B. Pediatric P-ANCA vasculitis following COVID-19. Pediatr Pulmonol. 2021; 56: 3422-3424.

29. Gaillard F, Ismael S, Sannier A, Tarhini H, Volpe T, Greze C, Verpont MC, Zouhry I, Rioux C, Lescure FX, Buob D, Daugas E. Tubuloreticular inclusions in COVID-19-related collapsing glomerulopathy. Kidney Int. 2020; 98: 241.

30. Gallagher MK, Dimitri DS, Wang D, Saha S. Kidney disease in the aftermath of COVID-19 infection. J Am Soc Nephrol. 2021; 766.

31. Gambella A, Barreca A, Biancone L, Roccatello D, Peruzzi L, Besso L, Licata C, Attanasio A, Papotti M, Cassoni P. Spectrum of kidney injury following COVID-19 disease: Renal biopsy findings in a single italian pathology service. Biomolecules. 2022; 12: 298.

32. Ganglam AB, Batool F, Miick R, Chewaproug D, Pedroza MA. A rare case of crescentic glomerulonephritis, diffuse proliferative class iv lupus nephritis, and collapsing glomerulopathy in a COVID, P-ANCA, and myeloperoxidase-positive patient. J Am Soc Nephrol. 2021; 505.

33. GARCIA-VEGA A, Santoyo-Fexas L, Maya-Quintá RD. POS-851 MPO-ANCA associated vasculitis after COVID-19. Kidney International Reports. 2022; 7: S367-368.

34. Shieh M, Giannini JA, Combs SA, Shaffi SK, Messias NC, Teixeira JP. Proliferative glomerulonephritis with monoclonal immunoglobulin deposits triggered by COVID-19: A case report. CEN Case Rep. 2022; 11: 380-385.

35. Goodman S, Kigonya C, Chow T, Bassil C. Patient with COVID-19 related collapsing glomerulopathy. Am J Kidney Dis. 2021; 598-599.

36. Guo W, Tan PH, Baikunje S. Membranous nephropathy in a patient with COVID-19 infection. J Nephrol. 2022; 35: 351-355.

37. Gupta RK, Bhargava R, Shaukat AA, Albert E, Leggat J. Spectrum of podocytopathies in new-onset nephrotic syndrome following COVID-19 disease: A report of 2 cases. BMC Nephrol. 2020; 21: 326.

38. Hale M, Barney EJ, Jean-Claude YD, Vanbeek CA. Collapsing FSGS in COVID-19. J Am Soc Nephrol 2020; 289.

39. Heylen L, Boeckx B, Jacobs T, Bosisio FM, Weynand B, Marcelis L, Dendooven A, Hendrickx L, Fransis S, Steensels D, Van Boxstael S. MO249 - Single-cell transcriptome of covid19 associated iga nephropathy. Nephrology Dialysis Transplantation. 2021; 36: gfab104-007.

40. Hoilat GJ, Das G, Shahnawaz M, Shanley P, Bukhari SH. COVID-19 induced collapsing glomerulopathy and role of APOL1. QJM. 2021; 114: 263-264.

41. Izci Duran T, Turkmen E, Dilek M, Sayarlioglu H, Arik N. ANCA-associated vasculitis after COVID-19. Rheumatol Int. 2021; 41: 1523-1529.

42. Izzedine H, Brocheriou I, Arzouk N, Seilhean D, Couvert P, Cluzel P, Pha M, Le Monnier O, Varnous S, Andreelli F, Amoura Z, Mathian A. COVID-19-associated collapsing glomerulopathy: A report of two cases and literature review. Intern Med J. 2020; 50: 1551-1558.

43. Jalalzadeh M, Valencia-Manrique JC, Boma N, Chaudhari A, Chaudhari S. Antineutrophil cytoplasmic antibody-associated glomerulonephritis in a case of scleroderma after recent diagnosis with COVID-19. Cureus. 2021; 13: e12485.

44. Bołtuć K, Bielejewska A, Coloma-Millar A, Dziugieł R, Bociek A, Perkowska-Ptasińska A, Jaroszyński A. Case Report: Cyclophosphamide in COVID-19 - when an absolute contraindication is an absolute necessity. F1000Res. 2021; 10: 829.

45. Jedlowski PM, Jedlowski MF. Coronavirus disease 2019-associated immunoglobulin A vasculitis/Henoch-Schönlein purpura: A case report and review. J Dermatol. 2022; 49: 190-196.

46. Kataria S, Rogers S, Sadia H, Ali T, Qureshi HM, Bano S, Anigbo CL, Singh R. Antineutrophil cytoplasmic antibody (ANCA)-associated renal vasculitis after COVID-19 infection: A case report. Cureus. 2022;14: e26111.

47. Kawashima S, Kishimoto M, Hibino T, Lee H, Sato Y, Komagata Y, Kaname S. MPO-ANCA-positive microscopic polyangiitis following COVID-19 infection. Intern Med. 2022; 61: 567-570.

48. Kazzi B, Fine D, Geetha D, Chung M, Monroy-Trujillo M, Timlin H. New-onset lupus nephritis associated with COVID-19 infection. Lupus. 2022; 31: 1007-1011.

49. Kesiena O, Papadopoulos P, Amakye D, Hama E, Mackay R. COVID-19 associated collapsing glomerulopathy presenting as acute kidney injury on chronic kidney disease: A case report and review of the literature. CEN Case Rep. 2022; 11: 273-277.

50. Khan J, Bukhari SW, Alam MG. Does SARS-cov-2 activate the alternative complement pathway A case report of c3-dominant proliferative GN in a patient with SARS-CoV-2 infection. J Am Soc Nephrol 2021; 466-467.

51. Kissling S, Rotman S, Gerber C, Halfon M, Lamoth F, Comte D, Lhopitallier L, Sadallah S, Fakhouri F. Collapsing glomerulopathy in a COVID-19 patient. Kidney Int. 2020; 98: 228-231.

52. Koc NS, Yildirim T, Saglam A, Arici M, Erdem Y. A patient with COVID-19 and anti-glomerular basement membrane disease. Nefrologia. 2021; 41: 471-473.

53. Kudose S, Santoriello D, Bomback AS, Sekulic M, Batal I, Stokes MB, Ghavami IA, Kim JS, Marasa M, Xu K, Peleg Y, Barasch J, Canetta P, Rasouly HM, Gharavi AG, Markowitz GS, D'Agati VD. Longitudinal Outcomes of COVID-19-Associated collapsing glomerulopathy and other podocytopathies. J Am Soc Nephrol. 2021; 32: 2958-2969.

54. Kulkarni A, Nasa P, Polumuru S, Singh A. Thrombotic microangiopathy causing acute kidney injury in a COVID-19 patient. Indian J Nephrol. 2021; 31: 559-561.

55. Laboux T, Gibier JB, Pottier N, Glowacki F, Hamroun A. Correction to: COVID-19-related collapsing glomerulopathy revealing a rare risk variant of APOL1: lessons for the clinical nephrologist. J Nephrol. 2021; 34: 379.

56. Larsen CP, Bourne TD, Wilson JD, Saqqa O, Sharshir MA. Collapsing glomerulopathy in a patient with COVID-19. Kidney Int Rep. 2020; 5: 935-939.

57. Lazoff SA, Chopra T, Abdel-Rahman EM. IGA vasculitis with renal manifestations in a college-aged individual after COVID-19 infection. J Am Soc Nephrol 2021; 90.

58. Leung C, McCormack G. Coronavirus-associated bronchiolitis in an immunocompetent adult with anti-glomerular basement membrane disease. Can. J. Respir. Crit. Care Sleep Med. 2019; 3: 117-120.

59. Li NL, Papini AB, Shao T, Girard L. Immunoglobulin-A vasculitis with renal involvement in a patient with COVID-19: A case report and review of acute kidney injury related to SARS-CoV-2. Can. j. kidney health dis. 2021; 8: 2054358121991684.

60. Lind E, Jameson A, Kurban E. Fulminant granulomatosis with polyangiitis presenting with diffuse alveolar haemorrhage following COVID-19. BMJ Case Rep. 2021; 14: e242628.

61. Madanchi N, Stingo FE, Patrick KC, Muthusamy S, Gupta N, Imran Fatani Y, Shah N. Possible association between COVID-19 infection and de novo antineutrophil cytoplasmic antibody-associated vasculitis. Cureus. 2021; 13: e20331.

62. Magoon S, Bichu P, Malhotra V, Alhashimi F, Hu Y, Khanna S, Berhanu K. COVID-19-related glomerulopathy: A report of 2 cases of collapsing focal segmental glomerulosclerosis. Kidney Med. 2020; 2: 488-492.

63. Maldonado D, Ray J, Lin X, Salem F, Brown M, Bansal I. COVAN Leading to ESKD despite minimal COVID symptoms. J Investig Med High Impact Case Rep. 2022; 10: 23247096221093888.

64. Malhotra V, Magoon S, Troyer DA, McCune TR. Collapsing focal segmental glomerulosclerosis and acute oxalate nephropathy in a patient with COVID-19: A Double Whammy. J Investig Med High Impact Case Rep. 2020; 8: 2324709620963635.

65. Malik IO, Ladiwala N, Chinta S, Khan M, Patel K. Severe acute respiratory syndrome coronavirus 2 induced focal segmental glomerulosclerosis. Cureus. 2020; 12: e10898.

66. Maritati F, Moretti MI, Nastasi V, Mazzucchelli R, Morroni M, Bagnarelli P, Rupoli S, Tavio M, Galiotta P, Bisello W, Ranghino A. ANCA-associated glomerulonephritis and anti-phospholipid syndrome in a patient with SARS-CoV-2 infection: Just a Coincidence? Case Rep Nephrol Dial. 2021; 11: 214-220.

67. May RM, Cassol C, Hannoudi A, Larsen CP, Lerma EV, Haun RS, Braga JR, Hassen SI, Wilson J, VanBeek C, Vankalakunti M, Barnum L, Walker PD, Bourne TD, Messias NC, Ambruzs JM, Boils CL, Sharma SS, Cossey LN, Baxi PV, Palmer M, Zuckerman JE, Walavalkar V, Urisman A, Gallan AJ, Al-Rabadi LF, Rodby R, Luyckx V, Espino G, Santhana-Krishnan S, Alper B, Lam SG, Hannoudi GN, Matthew D, Belz M, Singer G, Kunaparaju S, Price D, Chawla S, Rondla C, Abdalla MA, Britton ML, Paul S, Ranjit U, Bichu P, Williamson SR, Sharma Y, Gaspert A, Grosse P, Meyer I, Vasudev B, El Kassem M, Velez JCQ, Caza TN. A multi-center retrospective cohort study defines the spectrum of kidney pathology in Coronavirus 2019 disease (COVID-19). Kidney Int. 2021; 100: 1303-1315.

68. Mbengue M, Bigirimana B, Irankunda LR, Dial MC, Niang A. Recurrence or reactivation of SARS-CoV-2 infection after immunosuppressive therapy in patients with ANCA-associated vasculitis and COVID-19. Clin Nephrol Case Stud. 2022; 10: 6-10.

69. Meliambro K, Li X, Salem F, Yi Z, Sun Z, Chan L, Chung M, Chancay J, Vy HMT, Nadkarni G, Wong JS, Fu J, Lee K, Zhang W, He JC, Campbell KN. Molecular Analysis of the kidney from a patient with COVID-19-associated collapsing glomerulopathy. Kidney Med. 2021; 3: 653-658.

70. Miao J, Fidler ME, Nasr SH, Larsen CP, Zoghby ZM. Membranous nephropathy in a patient with coronavirus disease 2019 (COVID-19): A case report. Clin Nephrol Case Stud. 2021; 9: 11-18.

71. Moeinzadeh F, Dezfouli M, Naimi A, Shahidi S, Moradi H. Newly diagnosed glomerulonephritis during COVID-19 infection undergoing immunosuppression therapy, A case report. Iran J Kidney Dis. 2020; 14: 239-242.

72. Naqvi SM, Schwartz C, Whittaker R, Hammad G, Agarwal R. A rare case of mild COVID-19 disease associated with type 1 cryoglobulinemia and thrombotic thrombocytopenic purpura. Blood. 2021; 138: 4253.

73. Nasr SH, Alexander MP, Cornell LD, Herrera LH, Fidler ME, Said SM, Zhang P, Larsen CP, Sethi S. Kidney biopsy findings in patients with COVID-19, kidney injury, and proteinuria. Am J Kidney Dis. 2021; 77: 465-468.

74. Nlandu YM, Makulo JR, Pakasa NM, Sumaili EK, Nkondi CN, Bukabau JB, Beya FK, Nseka NM, Lepira FB. First case of COVID-19-associated collapsing glomerulopathy in Sub-Saharan Africa. Case Rep Nephrol. 2020; 2020: 8820713.

75. Noble R, Tan MY, McCulloch T, Shantier M, Byrne C, Hall M, Jesky M. Collapsing glomerulopathy affecting native and transplant kidneys in individuals with COVID-19. Nephron. 2020; 144: 589-594.

76. Nomura E, Finn LS, Bauer A, Rozansky D, Iragorri S, Jenkins R, Al-Uzri A, Richardson K, Wright M, Kung VL, Troxell ML, Andeen NK. Pathology findings in pediatric patients with COVID-19 and kidney dysfunction. Pediatr Nephrol. 2022; 37: 2375-2381.

77. Pasilan RM, Villanueva AR, Manalili SA. minimal change disease secondary to sars-cov-2 (covid-19) infection: A report of 2 cases and review of related literature. Nephrology Dialysis Transplantation. 2022; i139-140.

78. Peleg Y, Kudose S, D'Agati V, Siddall E, Ahmad S, Nickolas T, Kisselev S, Gharavi A, Canetta P. Acute kidney injury due to collapsing glomerulopathy following COVID-19 Infection. Kidney Int Rep. 2020; 5: 940-945.

79. Peracha J, Shah R, Neil D, Harper L, Chanouzas D. COVID-19 associated collapsing variant of focal segmental glomerulosclerosis: a case series of 7 patients from a single uk centre. Nephrology Dialysis Transplantation. 2022; i171-2.

80. Pérez A, Torregrosa I, D'Marco L, Juan I, Terradez L, Solís MÁ, Moncho F, Carda-Batalla C, Forner MJ, Gorriz JL. IgA-dominant infection-associated glomerulonephritis following SARS-CoV-2 infection. Viruses. 2021; 13: 587.

81. Román JL, Vergara A, Agraz I, García-Carro C, Bermejo S, Gabaldón A, Soler MJ. Focal and segmental glomerulosclerosis associated with COVID-19 infection. Nefrologia (Engl Ed). 2021; 41: 706-708.

82. Rosaly JP, Pabon-Vazquez E, Rivera BL. COVID-19-induced P-ANCA-associated nephritic syndrome in a woman without respiratory involvement. J Am Soc Nephrol. 2021; 93.

83. Rossi GM, Delsante M, Pilato FP, Gnetti L, Gabrielli L, Rossini G, Re MC, Cenacchi G, Affanni P, Colucci ME, Picetti E, Rossi S, Parenti E, Maccari C, Greco P, Di Mario F, Maggiore U, Regolisti G, Fiaccadori E. Kidney biopsy findings in a critically ill COVID-19 patient with dialysis-dependent acute kidney injury: A case against "SARS-CoV-2 nephropathy". Kidney Int Rep. 2020; 5: 1100-1105.

84. Roy S, Kunaparaju S, Koduri NM, Sangani V, Pokal M, Konala VM, Balla M, Adapa S. COVID-19 and APOL-1 High-risk genotype-associated collapsing glomerulonephritis. Case Rep Nephrol. 2021; 2021: 3737751.

85. Sandhu S, Chand S, Bhatnagar A, Dabas R, Bhat S, Kumar H, Dixit PK. Possible association between IgA vasculitis and COVID-19. Dermatol Ther. 2021; 34: e14551.

86. Sebastian R, Arunachalam J, Rajendran M. Temporal clustering of antiglomerular basement membrane disease in COVID-19 Pandemic: A Case Series. Int J Nephrol Renovasc Dis. 2021; 14: 393-398.

87. Selvaraj V, Moustafa A, Dapaah-Afriyie K, Birkenbach MP. COVID-19-induced granulomatosis with polyangiitis. BMJ Case Rep. 2021; 14: e242142.

88. Seshan SV, Salvatore S, Fyfe-Kirschner BS, Miick R, Siddiqui AA, Kahila M, Ramakrishnan R, Freundlich RE, Nicastri AD. COVID-19-associated nephropathy (COVAN): An emerging entity of severe viral podocyte injury and collapsing glomerulopathy in kidney biopsies. J Am Soc Nephrol. 2020; 298-299.

89. Sethi S, D'Costa MR, Hermann SM, Nasr SH, Fervenza FC. Immune-complex glomerulonephritis after COVID-19 Infection. Kidney Int Rep. 2021; 6: 1170-1173.

90. Sharma P, Uppal NN, Wanchoo R, Shah HH, Yang Y, Parikh R, Khanin Y, Madireddy V, Larsen CP, Jhaveri KD, Bijol V; Northwell Nephrology COVID-19 Research Consortium. COVID-19-associated kidney injury: A case series of kidney biopsy findings. J Am Soc Nephrol. 2020; 31: 1948-1958.

91. Sharma Y, Nasr SH, Larsen CP, Kemper A, Ormsby AH, Williamson SR. COVID-19-associated collapsing focal segmental glomerulosclerosis: A report of 2 cases. Kidney Med. 2020; 2: 493-497.

92. Sherchan S, Kahila M, Durrani JK, Puri I, Mohamed IA, Melaku Y, Yap E, Leonardo RF, Saggi SJ, Salifu MO, Nicastri AD. Collapsing/sclerosing glomerulopathy (CSG) and acute tubular injury (ATI) in patients with COVID-19. J Am Soc Nephrol. 2020; 279-280.

93. Sherchan S, Mohamed A, Vo T, D'Souza N, Clark D, Yap E, Tabriziani H. Acute kidney injury and collapsing glomerulopathy in patient with covid-19 and homozygous for apol1 gene variant. Am J Kidney Dis. 2021; 664-665.

94. Shetty AA, Tawhari I, Safar-Boueri ML, Seif N, Alahmadi AS, Aggarwal V, Gargiulo R, Kanwar YS, Quaggin SE. Covan, COVID-associated nephropathy: An evolving epidemic of kidney disease. J Am Soc Nephrol. 2020; 294.

95. Shieh M, Giannini JA, Combs SA, Shaffi SK, Messias NC, Teixeira JP. Proliferative glomerulonephritis with monoclonal immunoglobulin deposits triggered by COVID-19: a case report. CEN Case Rep. 2022; 11: 380-385.

96. Spinella KE, Motz RG. Treatment outcome of new-onset collapsing focal segmental glomerulosclerosis in a patient with COVID-19. Kidney week 2021 - poster. J Am Soc Nephrol. 2021; 32: 767.

97. Suso AS, Mon C, Oñate Alonso I, Galindo Romo K, Juarez RC, Ramírez CL, Sánchez Sánchez M, Mercado Valdivia V, Ortiz Librero M, Oliet Pala A, Ortega Marcos O, Herrero Berron JC, Silvestre Torner N, Alonso Riaño M, Pascual Martin A. IgA vasculitis with nephritis (Henoch-Schönlein Purpura) in a COVID-19 patient. Kidney Int Rep. 2020; 5: 2074-2078.

98. Tancredi T, DeWaters A, McGillen KL. Renal ultrasound findings secondary to COVID-19 related collapsing focal segmental glomerulosclerosis - A case report. Clin Imaging. 2021; 71: 34-38.

99. Thilakaratne D, Jenkinson P, Kwon K, Al-Shweiat W. COVID-19–associated nephrotic syndrome. Am J Kidney Dis. 2022; S48.

100. Uppal NN, Kello N, Shah HH, Khanin Y, De Oleo IR, Epstein E, Sharma P, Larsen CP, Bijol V, Jhaveri KD. De Novo ANCA-associated vasculitis with glomerulonephritis in COVID-19. Kidney Int Rep. 2020; 5: 2079-2083.

101. N V, Singh RKN, Kumari N, Ranjan R, Saini S. A Novel Association Between Coronavirus Disease 2019 and Normocomplementemic rapidly progressive glomerulonephritis-crescentic immunoglobulin A nephropathy: A report of two pediatric cases. Cureus. 2022; 14: e22077.

102. Waseem H, Zafar F, Anser M, Zia M. Pauci immune glomerulonephritis; A late complication of COVID-19 infection. InTP100. TP100 unexpected COVID-19 case reports 2021 May (pp. A4089-A4089). American Thoracic Society.

103. Wu H, Larsen CP, Hernandez-Arroyo CF, Mohamed MMB, Caza T, Sharshir M, Chughtai A, Xie L, Gimenez JM, Sandow TA, Lusco MA, Yang H, Acheampong E, Rosales IA, Colvin RB, Fogo AB, Velez JCQ. AKI and Collapsing glomerulopathy associated with COVID-19 and APOL1 high-risk genotype. J Am Soc Nephrol. 2020; 31: 1688-1695.

104. Yonishi H, Katada R, Kofune K, Kusunoki Y, Ikeda N, Ueno N, Teramoto K, Takeji M. New-onset immunoglobulin-A nephropathy post severe acute respiratory syndrome-coronavirus-2 infection indicates rapidly progressive glomerulonephritis. Nephrology (Carlton). 2022; 27: 542-543.

105. Zakrocka I, Korolczuk A, Załuska W. Antineutrophil cytoplasmic autoantibody-associated vasculitis with rapid progressive glomerulonephritis following SARS-CoV-2 infection: a cause or coincidence. Pol Arch Intern Med. 2022; 132: e242-245.

106. Zemke AM, Baxi PV, Rodby RA. Corticosteroid treatment in a case of COVID-19-associated collapsing FSGS. J Am Soc Nephrol. 2020; 286-287.

**Supplementary table 3:** Glomerular diseases following COVID-19 vaccination

| **Author, year** | **Ethnicity** | **Region** | **Age**  **(Years)** | **Sex** | **Vaccine type** | **Vaccine name** | **Vaccine frequency** | **Onset**  **(Days)** | **GD diagnosis** | **EM done** | **ATN** | **ATIN** | **TMA** | **Immunosuppressive treatment for GD** | **Treatment outcome** |
| --- | --- | --- | --- | --- | --- | --- | --- | --- | --- | --- | --- | --- | --- | --- | --- |
| Abdulgayoom et al., 2021^1^ | NA | Asia | 45 | F | mRNA | Pfizer | 1 | 4 | MCD | NA | NA | NA | NA | Yes | NA |
| Abramson et al., 2021^2^ | Hispanic/Latino | North America | 30 | M | mRNA | Moderna | 2 | 1 | IgAN | Yes | No | No | No | No | Complete remission |
| Acharya et al., 2021^3^ | NA | North America | 63 | F | mRNA | Pfizer | 2 | 3 | IgAN | NA | Yes | No | No | Yes | NA |
| Alhosaini et al., 2022^4^ | Caucasian | Asia | 16 | M | mRNA | Pfizer | 2 | 7 | MCD | Yes | NA | NA | NA | Yes | Partial remission |
| Al-Sawalmeh et al., 2022^5^ | NA | North America | 54 | F | mRNA | Pfizer | 2 | 7 | Crescentic GN - Fibrillary GN | Yes | Yes | No | No | Yes | No response |
| Baskaran et al., 2022^6^ | NA | Oceania | 31 | F | mRNA | unspecified | 2 | 21 | MCD | NA | NA | NA | NA | Yes | NA |
| Beynon et al., 2022^7^ | NA | Europe | 29 | F | mRNA | Pfizer | 1 | NA | LN type 3 | NA | NA | NA | NA | Yes | Complete remission |
|  | NA | Europe | 70 | F | Adenovirus-DNA | AstraZeneca | 1 | NA | LN type 3 | NA | NA | NA | NA | Yes | Responding to the treatment |
| Biradar et al., 2022^8^ | NA | Asia | 22 | M | Adenovirus-DNA | AstraZeneca | 1 | 16 | MCD | NA | NA | NA | NA | Yes | Complete remission |
| Caza et al., 2021^9^ | Hispanic/Latino | North America | 16 | F | mRNA | Pfizer | 1 | 2 | LN type not specified | Yes | No | No | No | Yes | Complete remission |
|  | Caucasian | North America | 23 | M | Adenovirus-DNA | AstraZeneca | 1 | 14 | MCD | Yes | Yes | No | No | Yes | Complete remission |
|  | African | North America | 26 | F | mRNA | Moderna | 2 | 6 | FSGS-collapsing glomerulopathy + MN-antigen not specified | Yes | Yes | No | No | Yes | No response |
|  | Caucasian | North America | 30 | M | mRNA | Pfizer | 2 | 1 | IgAN | Yes | Yes | No | No | NA | Complete remission |
|  | Caucasian | North America | 33 | F | mRNA | Pfizer | 2 | 2 | IgAN | Yes | No | No | No | No | NA |
|  | Caucasian | North America | 35 | M | mRNA | Pfizer | 2 | 1 | IgAN | Yes | Yes | No | No | Yes | No response |
|  | Caucasian | North America | 37 | F | mRNA | Unspecified | 2 | 12 | IgAN | Yes | Yes | No | No | Yes | No response |
|  | Caucasian | North America | 40 | F | mRNA | Moderna | 2 | 6 | IgAN | Yes | Yes | No | No | No | No response |
|  | Asian | North America | 43 | F | mRNA | Pfizer | 2 | 14 | MCD | Yes | Yes | No | No | Yes | Complete remission |
|  | Caucasian | North America | 45 | F | mRNA | Moderna | 1 | 13 | MCD | Yes | Yes | No | No | Yes | NA |
|  | Caucasian | North America | 47 | F | mRNA | Pfizer | 2 | 13 | MCD | Yes | Yes | No | No | Yes | No response |
|  | Asian | North America | 47 | M | mRNA | Moderna | 2 | 6 | MN-antigen not specified | Yes | No | No | No | NA | Partial remission |
|  | Caucasian | North America | 52 | F | mRNA | Unspecified | 2 | 1 | IgAN | Yes | No | No | No | NA | Complete remission |
|  | Asian | North America | 54 | M | mRNA | Moderna | 2 | 1 | MN-antigen not specified | Yes | Yes | No | No | Yes | No response |
|  | Caucasian | North America | 57 | M | mRNA | Moderna | 2 | 1 | IgAN | Yes | Yes | No | No | Yes | No response |
|  | Caucasian | North America | 65 | F | mRNA | Pfizer | 2 | 14 | Crescentic GN with positive ANCA-type unknown | Yes | Yes | No | No | Yes | No response |
|  | Hispanic/Latino | North America | 66 | M | mRNA | unspecified | 2 | 3 | IgAN | Yes | No | No | No | NA | No response |
|  | African | North America | 67 | F | mRNA | Moderna | 2 | 6 | FSGS-collapsing glomerulopathy | Yes | Yes | No | No | Yes | Partial remission |
|  | Caucasian | North America | 68 | M | Adenovirus-DNA | Janssen | 1 | 27 | MN-antigen not specified | Yes | Yes | No | No | No | Partial remission |
|  | Caucasian | North America | 70 | F | mRNA | Pfizer | 2 | 6 | MCD | Yes | Yes | No | No | Yes | Complete remission |
|  | Caucasian | North America | 71 | F | mRNA | Moderna | 2 | 14 | Crescentic GN with positive ANCA-type unknown | Yes | Yes | No | No | Yes | No response |
|  | Hispanic/Latino | North America | 72 | F | mRNA | Pfizer | 2 | 2 | IgAN | Yes | Yes | No | No | Yes | No response |
|  | Caucasian | North America | 72 | M | mRNA | Moderna | 2 | 7 | MCD | Yes | NA | No | No | Yes | Complete remission |
|  | Caucasian | North America | 73 | M | mRNA | Pfizer | 2 | 14 | IgAN | Yes | No | Yes | No | Yes | Partial remission |
|  | Caucasian | North America | 76 | F | mRNA | Moderna | 1 | 5 | Crescentic GN with positive ANCA-type unknown | Yes | Yes | No | No | Yes | Partial remission |
|  | Caucasian | North America | 76 | M | mRNA | Pfizer | 2 | 11 | Crescentic GN with positive ANCA-type unknown | Yes | No | No | No | Yes | No response |
|  | Asian | North America | 79 | M | mRNA | unspecfied | 1 | 13 | MCD | Yes | Yes | No | No | Yes | No response |
|  | Caucasian | North America | 81 | F | mRNA | Pfizer | 2 | 2 | Crescentic GN with positive ANCA-type unknown | Yes | Yes | No | No | Yes | No response |
| Chen et al., 2021^10^ | NA | Asia | 70 | F | mRNA | Moderna | 1 | 21 | AAV MPO | NA | NA | NA | NA | Yes | Responding to the treatment |
| Choi et al., 2022^11^ | NA | Asia | 74 | M | Adenovirus-DNA | AstraZeneca | 2 | 14 | IgAN | NA | NA | NA | NA | Yes | NA |
| Christodoulou et al., 2022^12^ | Caucasian | Europe | 72 | F | mRNA | Moderna | 2 | 15 | AAV-MPO | NA | No | No | No | Yes | Complete remission |
| Da et al., 2021^13^ | NA | Asia | 70 | M | mRNA | Pfizer | NA | NA | MN-THSD7A antibody positive | Yes | No | No | No | NA | No response |
| D'Agati et al., 2021^14^ | Caucasian | North America | 77 | M | mRNA | Pfizer | 1 | 16 | MCD | Yes | Yes | No | No | Yes | Partial remission |
| de Sa et al., 2022^15^ | NA | North America | 23 | F | mRNA | Pfizer | 2 | 2 | IgAN | NA | No | No | No | NA | NA |
| Dube et al., 2021^16^ | NA | North America | 23 | F | mRNA | Pfizer | 2 | 63 | AAV-MPO | Yes | No | No | No | Yes | Complete remission |
| El Hasbani et al., 2022^17^ | African | North America | 47 | F | mRNA | Pfizer | 1 | 3 | AAV-MPO | Yes | No | No | No | Yes | Complete remission |
| Feghali et al., 2021^18^ | Caucasian | North America | 58 | M | mRNA | Moderna | 2 | 60 | AAV-PR3 | NA | No | No | No | Yes | Complete remission |
| Fehr et al., 2021^19^ | NA | Europe | 65 | M | mRNA | Moderna | 1 | 8 | MCD | Yes | NA | NA | NA | Yes | Complete remission |
| Fernández et al., 2021^20^ | Hispanic/Latino | South America | 38 | F | Killed whole virus | CorV/Sinopham | 1 | 7 | IgAN | NA | No | Yes | No | Yes | Partial remission |
|  | Hispanic/Latino | South America | 53 | M | Adenovirus-DNA | AstraZeneca | 1 | 10 | IgAN | NA | No | Yes | No | Yes | Partial remission |
|  | Hispanic/Latino | South America | 59 | F | Adenovirus-DNA | Sputnik | 1 | 14 | MPGN | NA | No | No | No | Yes | Partial remission |
| Fornara et al., 2022^21^ | NA | Europe | 18 | M | mRNA | Pfizer | NA | 1 | IgAN | NA | NA | NA | NA | Yes | Partial remission |
|  | NA | Europe | 40 | M | mRNA | Pfizer | NA | 17 | MPGN | NA | NA | NA | NA | Yes | Partial remission |
|  | NA | Europe | 52 | F | mRNA | Pfizer | NA | 49 | MN-antigen not specified | NA | NA | NA | NA | No | Responding to the treatment |
|  | NA | Europe | 66 | F | mRNA | Pfizer | NA | 160 | MCD | NA | NA | NA | NA | Yes | Partial remission |
|  | NA | Europe | 67 | M | mRNA | Pfizer | NA | 22 | IgAN | NA | NA | NA | NA | Yes | Partial remission |
| Garcia et al., 2022^22^ | Caucasian | South America | 78 | F | Killed whole virus | Sinovac Biotech |  | 60 | AAV-PR3 | NA | Yes | No | No | Yes | Responding to the treatment |
| Garg et al., 2022^23^ | NA | North America | 50 | F | mRNA | Pfizer | 2 | 56 | MN-NELL1 positive | NA | NA | NA | NA | NA | Complete remission |
| Gupta et al., 2021^24^ | Hispanic/Latino | North America | 23 | M | mRNA | Moderna | 2 | 20 | Anti-GBM + AAV-MPO | NA | Yes | Yes | No | NA | NA |
|  | NA | North America | 77 | M | mRNA | unspecfied | NA | 4 | Atypical anti-GBM | Yes | No | No | No | NA | NA |
| Hakroush et al., 2021^25^ | Caucasian | Europe | 79 | F | mRNA | Pfizer | 2 | 21 | AAV-MPO | NA | Yes | Yes | No | Yes | Complete remission |
| Hanna et al., 2021a^26^ | Caucasian | North America | 17 | M | mRNA | Pfizer | 2 | 9 | IgAN | No | No | No | No | Yes | Responding to the treatment |
| Hanna et al., 2021b^27^ | NA | North America | 60 | M | mRNA | Pfizer | 1 | 56 | MCD | Yes | Yes | No | No | Yes | Complete remission |
| Hassani et al., 2021^28^ | NA | Asia | 55 | F | Adenovirus-DNA | AstraZeneca | 2 | NA | MPGN | NA | No | No | No | Yes | Responding to the treatment |
| Hellie et al., 2022^29^ | NA | North America | 55 | F | Adenovirus-DNA | AstraZeneca | 2 | 63 | Immune complex mediated GN - Full house GN - not lupus | Yes | No | No | No | Yes | Complete remission |
| Horino, 2022^30^ | Asian | Asia | 17 | M | mRNA | Pfizer | 2 | NA | IgAN | NA | No | No | No | Yes | NA |
| Ito et al., 2022^31^ | NA | Asia | 18 | M | mRNA | Pfizer | 1 | 1 | IgAN | NA | NA | NA | NA | Yes | Complete remission |
| Jha et al., 2022^32^ | Asian | Asia | 21 | M | Adenovirus-DNA | AstraZeneca | 1 | 12 | FSGS | Yes | NA | NA | NA | Yes | NA |
| Jhaveri et al., 2021^33^ | NA | North America | NA | NA | mRNA | Unspecified | NA | 6 | MCD | NA | No | No | No | NA | NA |
|  | NA | North America | NA | NA | mRNA | Unspecified | NA | 6 | MCD | NA | No | No | No | NA | NA |
|  | NA | North America | NA | NA | mRNA | Unspecified | NA | 6 | MCD | NA | No | No | No | NA | NA |
|  | NA | North America | NA | NA | mRNA | Unspecified | NA | 6 | MCD | NA | No | No | No | NA | NA |
|  | NA | North America | NA | NA | mRNA | Unspecified | NA | 6 | MN-PLA2R positive | NA | No | No | No | NA | NA |
| Kaghazian et al., 2022^34^ | NA | North America | 83 | M | mRNA | Pfizer | 1 | 14 | AAV-MPO | NA | NA | NA | NA | Yes | NA |
| Kim et al., 2022a^35^ | NA | Asia | 60 | F | Adenovirus-DNA | AstraZeneca | 2 | 60 | LN type 3 | Yes | No | No | No | Yes | Responding to the treatment |
| Kim et al., 2022b^36^ | NA | Asia | 16 | F | mRNA | Pfizer | 2 | 14 | crescentic GN - type unknown | Yes | No | Yes | No | Yes | Responding to the treatment |
| Kim et al., 2023^37^ | NA | Asia | 72 | F | Adenovirus-DNA | AstraZeneca | 3 | 30 | AAV-MPO | Yes | Yes | No | No | Yes | Responding to the treatment |
| Kim et al., 2022c^38^ | NA | Asia | 77 | F | mRNA | Pfizer | 2 | 30 | pauci-immune vasculitis-cause unknown | NA | No | No | No | Yes | NA |
| Klomjit et al., 2021a^39^ | NA | North America | 43 | F | mRNA | Moderna | 2 | 14 | IgAN | NA | No | Yes | No | NA | NA |
|  | NA | North America | 66 | F | mRNA | unspecfied | NA | 56 | IgAN | NA | Yes | No | No | NA | NA |
| Klomjit et al., 2021b^40^ | Caucasian | North America | 38 | M | mRNA | Pfizer | 2 | 14 | IgAN | NA | NA | NA | NA | Yes | No response |
|  | Caucasian | North America | 44 | M | mRNA | Moderna | 1 | 14 | IgAN-AIN | NA | NA | NA | NA | Yes | Responding to the treatment |
|  | Caucasian | North America | 50 | F | mRNA | Pfizer | 2 | 28 | MN-Nell-1 positive | NA | NA | NA | NA | NA | Responding to the treatment |
|  | Caucasian | North America | 62 | M | mRNA | Pfizer | 2 | 42 | IgAN | NA | NA | NA | NA | NA | No response |
|  | Caucasian | North America | 66 | M | mRNA | Moderna | 1 | 14 | IgAN | NA | NA | NA | NA | Yes | Responding to the treatment |
|  | Caucasian | North America | 77 | M | mRNA | Pfizer | 1 | 7 | Anti-GBM - atypical | NA | NA | NA | NA | Yes | Responding to the treatment |
|  | Caucasian | North America | 82 | F | mRNA | Moderna | 2 | 28 | AAV-MPO | NA | NA | NA | NA | Yes | Responding to the treatment |
|  | Caucasian | North America | 83 | M | mRNA | Moderna | 2 | 28 | MCD | NA | NA | NA | NA | Yes | Responding to the treatment |
| Kobyashi et al., 2021^41^ | NA | Asia | 75 | M | mRNA | Pfizer | 2 | 9 | MCD | Yes | NA | NA | NA | Yes | Complete remission |
| Lebedev et al., 2021^42^ | NA | Asia | 50 | M | mRNA | Pfizer | 1 | 13 | MCD | Yes | Yes | No | No | Yes | Partial remission |
| Leclerc et al., 2021^43^ | NA | North America | 71 | M | Adenovirus-DNA | AstraZeneca | 1 | 17 | MCD | NA | No | No | No | Yes | Responding to the treatment |
| Lim et al., 2022a^44^ | NA | Asia | 24 | M | Killed whole virus | Sinovac Biotech | 1 | 17 | MCD | NA | NA | NA | NA | Yes | Complete remission |
| Lim et al., 2022b^45^ | NA | Asia | 42 | F | mRNA | Moderna | 2 | 54 | IgAN | Yes | No | No | No | No | Partial remission |
|  | NA | Asia | 52 | M | Adenovirus-DNA | Janssen | 1 | 33 | MCD | Yes | No | No | No | Yes | Complete remission |
| Lim et al., 2022c^46^ | NA | Asia | 29 | M | mRNA | Pfizer | 1 | 31 | FSGS | Yes | NA | NA | NA | Yes | Partial remission |
| Marega et al., 2022^47^ | Caucasian | Europe | 80 | F | mRNA | Pfizer | 2 | 60 | FSGS-collapsing glomerulopathy | NA | No | No | No | Yes | Complete remission |
| Marinaki et al., 2022^48^ | NA | Europe | 55 | F | mRNA | Pfizer | 1 | 4 | MCD | Yes | NA | NA | NA | Yes | Complete remission |
| Marouco et al., 2022^49^ | Caucasian | Europe | 62 | M | mRNA | Pfizer | 2 | 14 | AAV-MPO | Yes | No | No | No | Yes | Partial remission |
| Mohamed et al., 2021^50^ | NA | North America | 50 | M | mRNA | Pfizer | 2 | 28 | IgAN | Yes | No | No | No | Yes | Complete remission |
| Nakatani et al., 2022^51^ | NA | Asia | 47 | M | mRNA | unspecified | 2 | 29 | IgAN | Yes | No | Yes | No | Yes | Responding to the treatment |
| Neves et al., 2022^52^ | NA | South America | 58 | F | Adenovirus-DNA | AstraZeneca | 2 | 42 | FSGS-collapsing glomerulopathy | NA | Yes | No | No | NA | Responding to the treatment |
|  | African | South America | 63 | F | Adenovirus-DNA | AstraZeneca | 1 | 21 | FSGS-collapsing glomerulopathy | NA | Yes | No | No | Yes | Responding to the treatment |
| Obata et al., 2021^53^ | Asian | Asia | 84 | M | mRNA | Pfizer | 2 | 28 | AAV-MPO | Yes | NA | NA | NA | Yes | Responding to the treatment |
| Okada et al., 2022^54^ | NA | Asia | 17 | F | mRNA | Pfizer | 2 | 14 | IgAN | Yes | No | No | No | NA | Complete remission |
| Park et al., 2022^55^ | NA | Asia | 34 | M | mRNA | Moderna | 2 | 7 | MCD | Yes | NA | NA | NA | Yes | Partial remission |
|  | Asian | Asia | 60 | M | mRNA | Moderna | 2 | 9 | MCD | Yes | NA | NA | NA | Yes | Complete remission |
| Paxton et al., 2022^56^ | Caucasian | Oceania | 22 | M | mRNA | unspecified | 2 | 30 | MN-PLA2R positive | NA | No | No | No | Yes | Partial remission |
| Pella et al., 2022^57^ | Caucasian | Europe | 18 | M | mRNA | Pfizer | 1 | 30 | MCD | NA | NA | NA | NA | Yes | Complete remission |
| Perea-Ortega et al., 2022^58^ | NA | Europe | NA | NA | mRNA | unspecified | NA | NA | IgAN | NA | NA | NA | NA | NA | NA |
| Prabhahar et al., 2022^59^ | NA | Asia | 51 | M | Adenovirus-DNA | AstraZeneca | 1 | 15 | AAV-PR3 | NA | No | No | No | Yes | Responding to the treatment |
| Prema et al., 2021^60^ | NA | Asia | 45 | M | Recombinant protein based | Covax-19 | 1 | NA | Anti GBM + AAV-MPO | NA | NA | NA | NA | Yes | Responding to the treatment |
|  | NA | Asia | 58 | M | Recombinant protein based | Covax19 | 2 | NA | Anti-GBM + AAV-cANCA | NA | NA | NA | NA | Yes | Responding to the treatment |
| Ran et al., 2022^61^ | NA | Asia | 58 | M | Killed whole virus | Sinovac Biotech | 1 | 30 | IgAN-crescentic | NA | No | No | No | Yes | NA |
| Rashid et al., 2022^62^ | NA | North America | 56 | M | mRNA | Moderna | 1 | 30 | MN-PLA2R positive | NA | Yes | Yes | No | No | Partial remission |
| Ritter et al., 2021^63^ | Caucasian | Europe | 20 | M | mRNA | Moderna | 2 | NA | IgAN | NA | NA | NA | NA | No | Complete remission |
|  | Caucasian | Europe | 25 | F | mRNA | Pfizer | 2 | NA | IgAN | NA | NA | NA | NA | No | Partial remission |
|  | Asian | Europe | 37 | F | mRNA | Moderna | 2 | NA | IgAN | NA | NA | NA | NA | No | Partial remission |
|  | Caucasian | Europe | 69 | M | mRNA | Pfizer | 2 | NA | IgAN + AAV - ANCA type unknown | NA | NA | NA | NA | Yes | No response |
| Roberts et al., 2021^64^ | Caucasian | North America | 22 | F | mRNA | Pfizer | 2 | NA | IgAN | Yes | No | No | No | No | Partial remission |
| Saigal et al., 2022^65^ | NA | Asia | 24 | F | Adenovirus-DNA | AstraZeneca | NA | 7 | LN type 4 | NA | NA | NA | NA | Yes |  |
|  | NA | Asia | 32 | M | Adenovirus-DNA | AstraZeneca | NA | 14 | MN-antigen not specified | NA | NA | NA | NA | Yes | Partial remission |
|  | NA | Asia | 34 | M | Adenovirus-DNA | AstraZeneca | NA | 10 | MCD | NA | NA | NA | NA | Yes | Partial remission |
|  | NA | Asia | 47 | M | Adenovirus-DNA | AstraZeneca | NA | 11 | MN-antigen not specified | NA | NA | NA | NA | NA | Complete remission |
|  | NA | Asia | 52 | M | Killed whole virus | Covaxin | NA | 6 | AAV-ANCA type unknown | NA | NA | NA | NA | Yes | Responding to the treatment |
| Salem et al., 2021^66^ | NA | North America | 41 | F | mRNA | Pfizer | 2 | 5 | MCD | Yes | NA | NA | NA | NA | NA |
| Schaubsch et al., 2022^67^ | NA | North America | 35 | F | mRNA | Pfizer | 2 | 196 | IgAN | NA | NA | NA | NA | Yes | Complete remission |
|  | NA | North America | 60 | F | mRNA | Moderna | 2 | 31 | Immune complex mediated GN | NA | No | Yes | No | Yes | Complete remission |
|  | NA | North America | 77 | F | mRNA | Moderna | 2 | 105 | AAV-MPO | NA | NA | NA | NA | Yes | Partial remission |
| Seif et al., 2021^68^ | NA | North America | 66 | M | mRNA | Moderna | 2 | 21 | AAV-pANCA | NA | NA | NA | NA | Yes | Partial remission |
| Sekar et al., 2021^69^ | Caucasian | North America | 52 | M | mRNA | Moderna | 2 | NA | AAV-PR3 | NA | Yes | Yes | No | Yes | NA |
| Shakoor et al., 2021^70^ | NA | North America | 78 | F | mRNA | Pfizer | 1 | 16 | AAV-MPO | NA | No | Yes | No | Yes | Responding to the treatment |
| Sirpal et al., 2022^71^ | Caucasian | North America | 26 | M | mRNA | Moderna | 2 | 21 | FSGS-non-collapsing-tip variant | Yes | NA | NA | NA | Yes | Responding to the treatment |
| So et al., 2022^72^ | NA | Asia | 42 | M | mRNA | Pfizer | 2 | 29 | AAV-MPO | NA | No | No | No | Yes | Responding to the treatment |
| Srinivasan et al., 2022^73^ | Caucasian | North America | 35 | M | mRNA | Unspecified | 2 | 2 | IgAN | NA | NA | NA | NA | Yes | Partial remission |
| Sugita et al., 2022^74^ | Asian | Asia | 67 | F | mRNA | Pfizer | 2 | 14 | IgAN | Yes | No | No | No | Yes | Responding to the treatment |
| Tan et al., 2021^75^ | NA | Asia | 60 | F | mRNA | Pfizer | 2 | NA | Anti-GBM | NA | No | No | No | Yes | Responding to the treatment |
| Thappy et al., 2021^76^ | African | Aisa | 43 | M | mRNA | Moderna | 1 | 21 | MCD | NA | No | No | No | Yes | Responding to the treatment |
| Timmermans et al., 2022^77^ | NA | Europe | 34 | M | mRNA | Pfizer | 2 | 28 | MCD | NA | NA | NA | NA | NA | NA |
|  | NA | Europe | 47 | M | mRNA | Pfizer | 2 | 13 | FSGS- type not specified | NA | NA | NA | NA | NA | NA |
|  | NA | Europe | 64 | F | Adenovirus-DNA | AstraZeneca | 1 | 7 | MCD | NA | NA | NA | NA | NA | NA |
|  | NA | Europe | 74 | M | mRNA | Pfizer | 2 | 42 | MCD | NA | NA | NA | NA | NA | NA |
| Unver et al., 2021^78^ | NA | Asia | 67 | F | Killed whole virus | Sinovac Biotech | 2 | 28 | MCD | NA | No | Yes | No | Yes | Responding to the treatment |
| Villa et al., 2021^79^ | NA | Europe | 63 | M | Adenovirus-DNA | AstraZeneca | 1 | 7 | AAV-MPO | NA | No | No | No | Yes | Responding to the treatment |
| Yadav et al., 2022^80^ | NA | Asia | 52 | F | Adenovirus-DNA | Janssen | NA | 22 | AAV-cANCA and pANCA positive | NA | No | No | No | Yes | NA |
| Yokote et al., 2022^81^ | NA | Asia | 19 | M | mRNA | Pfizer | 2 | 30 | IgAN | NA | No | No | No | Yes | Responding to the treatment |
|  | NA | Asia | 36 | F | mRNA | Pfizer | 1 | 25 | IgAN | Yes | No | No | No | Yes | Responding to the treatment |
| Zavala-Miranda et al., 2021^82^ | NA | North America | 23 | F | Adenovirus-DNA | AstraZeneca | 1 | 14 | LN type 5 | Yes | No | No | No | Yes | Responding to the treatment |

COVID-19 = Coronavirus Disease 2019, GD = Glomerular Disease, M=Male, F=Female EM = Electron microscopy, ATN = Acute tubular necrosis, ATIN = Acute tubulointerstitial nephritis, TMA = Thrombotic microangiopathy, FSGS = Focal Segmental Glomerulosclerosis, MCD = Minimal Change Disease, IgAN = Immunoglobulin A Nephropathy, HSP = Henoch, Schoenlein purpura, HUS = Haemolytic uremic syndrome,, MPGN = Membranoproliferative Glomerulonephritis, LN = Lupus Nephritis, Anti-GBM = Anti glomerular membrane, GN = Glomerulonephritis, AAV = Anti-neutrophil cytoplasmic antibodies Associated Vasculitis, MPO = myeloperoxidase, PR3 = Proteinase 3, C3GN = Complement 3 Glomerulonephritis, PGNMID = Proliferative GN with Monoclonal Immunoglobulin Deposition, mRNA = messenger ribonucleic acid, NA = not available.

**References**

1. Abdulgayoom M, Albuni MK, Abdelmahmuod E, Murshed K, Eldeeb Y. Minimal change nephrotic syndrome four days after the administration of Pfizer-BioNTech COVID-19 vaccine-a new side effect or coincidence? Clin Case Rep. 2021; 23: 9e05003.

2. Abramson M, Yu SM, Campbell KN, Chung M, Salem F. IgA nephropathy after SARS-CoV-2 vaccination. Kidney Med. 2021; 3: 860-863.

3. Acharya PC, Acharya C, Medaura JA. IgA nephropathy post COVID-19 vaccination. J Am Soc Nephrol. 2021; 89.

4. Alhosaini MN. A case of minimal change disease after SARS-CoV-2 vaccination under the age of 18. Avicenna J Med. 2022; 12: 31-33.

5. Al-Sawalmeh K, Pandes M, Niño JA, Avila-Casado C. Acute kidney injury after Pfizer COVID-19 vaccine due to crescentic fibrillary glomerulonephritis. Clin Nephrol. 2022; 98: 205-208.

6. Baskaran K, Cohen AWS, Weerasinghe N, Vilayur E. Report of two cases of minimal change disease following vaccination for COVID -19. Nephrology (Carlton). 2022; 27: 111-112.

7. Beynon J, Alsharkawy M, Sammut L, Ledingham J. New-onset systemic lupus erythematosus following covid-19 vaccination: A report of two cases. Rheumatology. 2022; i131-2.

8. Biradar V, Konnur A, Gang S, Hegde U, Rajapurkar M, Patel H, Pandey SN, Soni S. Adult-onset nephrotic syndrome following coronavirus disease vaccination. Clin Kidney J. 2021; 15: 168-170.

9. Caza TN, Cassol CA, Messias N, Hannoudi A, Haun RS, Walker PD, May RM, Seipp RM, Betchick EJ, Amin H, Ziadie MS, Haderlie M, Eduwu-Okwuwa J, Vancea I, Seek M, Elashi EB, Shenoy G, Khalillullah S, Flaxenburg JA, Brandt J, Diamond MJ, Frome A, Kim EH, Schlessinger G, Ulozas E, Weatherspoon JL, Hoerschgen ET, Fabian SL, Bae SY, Iqbal B, Chouhan KK, Karam Z, Henry JT, Larsen CP. Glomerular disease in temporal association with SARS-CoV-2 vaccination: A series of 29 cases. Kidney360. 2021; 2: 1770-1780.

10. Chen CC, Chen HY, Lu CC, Lin SH. Case Report: Anti-neutrophil cytoplasmic antibody-associated vasculitis with acute renal failure and pulmonary hemorrhage may occur after COVID-19 vaccination. Front Med (Lausanne). 2021; 8: 765447.

11. Choi Y, Lee CH, Kim KM, Yoo WH. Sudden onset of IgA vasculitis affecting vital organs in adult patients following SARS-CoV-2 vaccines. 2022; 10 :923.

12. Christodoulou M, Iatridi F, Chalkidis G, Lioulios G, Nikolaidou C, Badis K, Fylaktou A, Papagianni A, Stangou M. ANCA-associated vasculitis may result as a complication to both SARS-CoV-2 infection and vaccination. 2022; 12: 1072.

13. Da Y, Goh GH, Khatri P. A case of membranous nephropathy following Pfizer-BioNTech mRNA vaccination against COVID-19. Kidney Int. 2021; 100: 938-939.

14. D'Agati VD, Kudose S, Bomback AS, Adamidis A, Tartini A. Minimal change disease and acute kidney injury following the Pfizer-BioNTech COVID-19 vaccine. Kidney Int. 2021;100: 461-463.

15. de Sa PN, Molovic-Kokovic A. De novo IgA nephropathy following mRNA COVID-19 Vaccine. Am J of Kidney Dis. 2022; S 65-66.

16. Dube GK, Benvenuto LJ, Batal I. Antineutrophil cytoplasmic autoantibody-associated glomerulonephritis following the Pfizer-BioNTech COVID-19 vaccine. Kidney Int Rep. 2021; 6: 3087-3089.

17. El Hasbani G, Uthman I. ANCA-associated vasculitis following the first dose of Pfizer-BioNTech COVID-19 vaccine. Nephron. 2023;147: 103-107.

18. Feghali EJ, Zafar M, Abid S, Santoriello D, Mehta S. De-novo antineutrophil cytoplasmic antibody-associated vasculitis following the mRNA-1273 (Moderna) vaccine for COVID-19. Cureus. 2021;13 :e19616.

19. Fehr N, Berney-Meyer L, Hopfer H, Räz HR. Minimal change disease (MCD) after Moderna COVID-19 vaccination. Swiss Medical Weekly. 2021; 35S.

20. Fernández P, Alaye ML, Chiple MEG, Arteaga J, Douthat W, Fuente J, Chiurchiu C. Glomerulopathies after vaccination against COVID-19: Four cases with three different vaccines in Argentina. Nefrologia (Engl Ed). 2022; S2013-2514: 00123-7.

21. Fornara L, Musetti C, Guglielmetti G, Cantaluppi V. De novo glomerulonephritides following BNT162B2 COVID-19 vaccine: A case series. Nephrology, dialysis, transplantation: official publication of the European Dialysis and Transplant Association-European Renal Association. 2022.

22. Garcia DS, Martins C, da Fonseca EO, de Carvalho VCP, de Rezende RPV. Clinical Images: Severe proteinase 3 antineutrophil cytoplasmic antibody glomerulonephritis temporally associated with Sinovac Biotech's inactivated SARS-CoV-2 vaccine. ACR Open Rheumatol. 2022; 4: 277-278.

23. Garg A. POS-897 a case of de novo nell-1 membranous nephropathy post covid-19 vaccination. Kidney Int Rep. 2022; 7: S389.

24. Gupta RK, Ellis BK. Concurrent antiglomerular basement membrane nephritis and antineutrophil cytoplasmic autoantibody-mediated glomerulonephritis after second dose of SARS-CoV-2 mRNA vaccination. Kidney Int Rep. 2022; 7: 127-128.

25. Hakroush S, Tampe B. Case Report: ANCA-associated vasculitis presenting with rhabdomyolysis and pauci-immune crescentic glomerulonephritis after Pfizer-BioNTech COVID-19 mRNA vaccination. Front Immunol. 2021; 12: 762006.

26. Hanna C, Herrera Hernandez LP, Bu L, Kizilbash S, Najera L, Rheault MN, Czyzyk J, Kouri AM. IgA nephropathy presenting as macroscopic hematuria in 2 pediatric patients after receiving the Pfizer COVID-19 vaccine. Kidney Int. 2021; 100: 705-706.

27. Hanna J, Ingram A, Shao T. Minimal change disease after first dose of Pfizer-BioNTech COVID-19 vaccine: A case report and review of minimal change disease related to COVID-19 vaccine. Can J Kidney Health Dis. 2021; 8: 20543581211058271.

28. Hassani K, Errihani M, Mahamoud MH, ElKabbaj D. Membranoproliferative glomerulonephritis following the Oxford AstraZeneca COVID-19 vaccine. Saudi J Kidney Dis Transpl. 2021; 32: 1831-1832.

29. Hellie K, Bu L, Klomjit N. Seronegative full-house immune complex glomerulonephritis post-mRNA COVID-19 vaccination. Am J Kidney Dis. 2022; S37.

30. Horino T, Sawamura D, Inotani S, Ishihara M, Komori M, Ichii O. Newly diagnosed IgA nephropathy with gross haematuria following COVID-19 vaccination. QJM. 2022; 115: 28-29.

31. Ito C, Odajima K, Niimura Y, Fujii M, Sone M, Asakawa S, Arai S, Yamazaki O, Tamura Y, Saito K, Tada Y, Yamamoto T, Kozuma K, Shibata S, Fujigaki Y. IgA vasculitis with transient glomerular hematuria, diarrhea, and pericarditis following COVID-19 mRNA vaccination in a young patient with possible pre-existing ulcerative colitis. CEN Case Rep. 2023;12: 84-90.

32. Jha VK, Akal RS, Sharma A, Mahapatra D. Post Covishield (ChAdOx1 nCoV-19) Vaccination: New onset focal segmental glomerulosclerosis resistant to steroid and calcineurin inhibitor. Indian J Nephrol. 2022; 32: 378-383.

33. Jhaveri KD, Bijol V, Wanchoo R, Keller KH, Alexander MP, Weins A. COVID-19 mRNA vaccine-associated autoimmunity presenting as minimal change disease and membranous nephropathy. J. Am. Soc. Nephrol. 2021; 94.

34. Kaghazian M, Akabusi CW, Acosta KP, Kallakuri K, Shakouri P. Anti-neutrophil cytoplasmic antibody associated vasculitis secondary to Pfizer-BioNTech COVID-19 vaccine. InA36. More to the story: case reports in connective tissue disease associated interstitial lung disease 2022; pp. A1384-A1384. Ann Am Thorac Soc.

35. Kim BC, Kim HS, Han KH, Han SY, Jo HA. A case report of mpo-anca-associated vasculitis following heterologous mRNA1273 COVID-19 booster vaccination. J Korean Med Sci. 2022; 37: e204.

36. Kim HJ, Jung M, Lim BJ, Han SH. New-onset class III lupus nephritis with multi-organ involvement after COVID-19 vaccination. Kidney Int. 2022; 101: 826-828.

37. Kim S, Jung J, Cho H, Lee J, Go H, Lee JH. A child with crescentic glomerulonephritis following SARS-CoV-2 mRNA (Pfizer-BioNTech) vaccination. Pediatr Nephrol. 2023; 38: 299-302.

38. Kim Y, Kang J, Lee SG, Kim GT. COVID-19 vaccination-related small vessel vasculitis with multiorgan involvement. Z Rheumatol. 2022; 81: 509-512.

39. Klomjit N, Alexander MP, Fervenza FC, Zoghby Z, Garg A, Hogan MC, Nasr SH, Minshar MA, Zand L. COVID-19 vaccination and glomerulonephritis. Kidney Int Rep. 2021; 6: 2969-2978.

40. Klomjit N, Zoghby Z, Fervenza FC, Zand L. New diagnosis of glomerulonephritis and relapse of prior glomerulonephritis post mRNA COVID-19 vaccination. J. Am. Soc. Nephrol. 2021; 90.

41. Kobayashi S, Fugo K, Yamazaki K, Terawaki H. Minimal change disease soon after Pfizer-BioNTech COVID-19 vaccination. Clin Kidney J. 2021; 14: 2606-2607.

42. Lebedev L, Sapojnikov M, Wechsler A, Varadi-Levi R, Zamir D, Tobar A, Levin-Iaina N, Fytlovich S, Yagil Y. Minimal change disease following the Pfizer-BioNTech COVID-19 vaccine. Am J Kidney Dis. 2021; 78: 142-145.

43. Leclerc S, Royal V, Lamarche C, Laurin LP. Minimal change disease with severe acute kidney injury following the Oxford-AstraZeneca COVID-19 vaccine: A case report. Am J Kidney Dis. 2021; 78: 607-610.

44. Lim CA, Lee HS, Yoon S, Kim EJ, Seo JW, Koo JR, Baek SH. Focal segmental glomerulosclerosis following the Pfizer-BioNTech COVID-19 vaccine. Kidney Res Clin Pract. 2022; 41: 263-266.

45. Lim JH, Kim MS, Kim YJ, Han MH, Jung HY, Choi JY, Cho JH, Kim CD, Kim YL, Park SH. New-onset kidney diseases after COVID-19 vaccination: A case series. 2022; 10: 302.

46. Lim YS, Leong CM, Nge CS, Mohamad H. Pos-134 - New onset nephrotic syndrome following inactivated sars-cov-2 vaccine. Kidney Int Rep. 2022; 7: S58.

47. Marega A, Pizzolitto S, Pian M, Biasi C, Giacomini A, Bertoni M, Romano G, Boscutti G. De novo double glomerulopathy (membranous nephropathy, mn and collapsing focal segmental glomerulosclerosis, cfsgs) associated to positive myeloperoxidase-o (mpo) antibody following pfizer-biontech mrna vaccination COVID 19. Nephrol. Dial*. Transplant.* 2022; i156-157.

48. Marinaki S, Kolovou K, Liapis G, Skalioti C, Tsiakas S, Boletis I. De novo minimal change disease following vaccination with the Pfizer/BioNTech SARS-CoV-2 Vaccine in a living kidney donor. 2021; 58: 37.

49. Marouco C, Carvalho D, Magriço R, Ribeiro F, Nolasco F. Anca-associated vasculitis following pfizer-biontech covid-19 vaccine: true association or circumstantial?. Nephrol. Dial. Transplant*.* 2022; i155.

50. Mohamed MMB, Wickman TJ, Fogo AB, Velez JCQ. De novo immunoglobulin A vasculitis following exposure to SARS-CoV-2 immunization. Ochsner J. 2021; 21: 395-401.

51. Nakatani S, Mori K, Morioka F, Hirata C, Tsuda A, Uedono H, Ishimura E, Tsuruta D, Emoto M. New-onset kidney biopsy-proven IgA vasculitis after receiving mRNA-1273 COVID-19 vaccine: Case report. CEN Case Rep. 2022; 11: 358-362.

52. Neves PD, Caires RA, Guimarães MP, Costalonga EC, Cavalcante LB, Costa E Silva VT, Mattedi FZ, Santana LF, Teixeira-Júnior AA, Gomes OV, Silva GE, Burdmann EA, Onuchic LF. Collapsing glomerulopathy following SARS-CoV-2 adenovirus-vector-based vaccine: Report of 2 cases. Kidney Int. 2022; 101: 637-639.

53. Obata S, Hidaka S, Yamano M, Yanai M, Ishioka K, Kobayashi S. MPO-ANCA-associated vasculitis after the Pfizer/BioNTech SARS-CoV-2 vaccination. Clin Kidney J. 2021; 15: 357-359.

54. Okada M, Kikuchi E, Nagasawa M, Oshiba A, Shimoda M. An adolescent girl diagnosed with IgA nephropathy following the first dose of the COVID-19 vaccine. CEN Case Rep. 2022; 11: 376-379.

55. Park HJ, An WS, Rha SH, Kim SE, Lee SM. Minimal change glomerulonephritis following the second dose of the Moderna COVID-19 vaccine. QJM. 2022; 115: 490-491.

56. Paxton L, McMahon L, Wong L. De novo PLA2R positive membranous nephropathy following BNT162b2 mRNA COVID-19 vaccine. Intern Med J. 2022; 52: 2191-2192.

57. Pella E, Sarafidis PA, Alexandrou ME, Stangou M, Nikolaidou C, Kosmidis D, Papagianni A. De novo minimal change disease in an adolescent after Pfizer-BioNTech COVID-19 vaccination: A case report. Case Rep Nephrol Dial. 2022; 12: 44-49.

58. Perea-Ortega L, Polo Criado C, Guzman PH, Sanchez ML, Rojas RT. Glomerulopathies after covid-19 vaccinationL: Report of cases in our centre. Nephrol. Dial. Transplant*.* 2022; i152.

59. Prabhahar A, Naidu GSRSNK, Chauhan P, Sekar A, Sharma A, Sharma A, Kumar A, Nada R, Rathi M, Kohli HS, Ramachandran R. ANCA-associated vasculitis following ChAdOx1 nCoV19 vaccination: case-based review. Rheumatol Int. 2022; 42: 749-758.

60. Prema J, Muthukumaran A, Haridas N, Fernando E, Seshadri J, Kurien AA. Two cases of double-positive antineutrophil cytoplasmic autoantibody and antiglomerular basement membrane disease after BBV152/Covaxin vaccination. Kidney Int Rep. 2021; 6: 3090-3091.

61. Ran E, Wang M, Wang Y, Liu R, Yi Y, Liu Y. New-onset crescent IgA nephropathy following the CoronaVac vaccine: A case report. Medicine (Baltimore). 2022; 101: e30066.

62. Rashid W, Mousa H, Khan J, Ijaz F, Ezell GD. A case of membranous nephropathy hypothesized to be associated with COVID-19 vaccine. Cureus. 2022; 14: e24245.

63. Ritter A, Helmchen B, Gaspert A, Bleisch J, Fritschi B, Buchkremer F, Damm S, Schmid N, Schachtner T, Seeger H. Clinical spectrum of gross haematuria following SARS-CoV-2 vaccination with mRNA vaccines. Clin Kidney J. 2021; 15: 961-973.

64. Roberts LL. Iga nephropathy after receiving the pfizer COVID-19 vaccine: A case report. J. Am. Soc. Nephrol. 2021; 89-90.

65. Saigal M, Taduri G, Gudditi S, Herur S, Alaparthi P, Kinjarapu S. POS-872. Post covid vaccination-new onset glomerulonephritis-a mere co incidence or a impending reality?. Kidney Int. Rep. 2022; 7: S377.

66. Salem F, Rein JL, Yu SM, Abramson M, Cravedi P, Chung M. Report of three cases of minimal change disease following the second dose of mRNA SARS-CoV-2 COVID-19 Vaccine. Kidney Int Rep. 2021; 6: 2523-2524.

67. Schaubschlager T, Rajora N, Diep S, Kirtek T, Cai Q, Hendricks AR, Shastri S, Zhou XJ, Saxena R. De novo or recurrent glomerulonephritis and acute tubulointerstitial nephritis after COVID-19 vaccination: A report of six cases from a single center. Clin Nephrol. 2022; 97: 289-297.

68. Seif N, Ellis CL, Wadhwani S. ANCA-associated glomerulonephritis and vasculitis following COVID-19 vaccination in a patient with giant cell arteritis. J. Am. Soc. Nephrol. 2021; 87.

69. Sekar A, Campbell R, Tabbara J, Rastogi P. ANCA glomerulonephritis after the Moderna COVID-19 vaccination. Kidney Int. 2021; 100: 473-474.

70. Shakoor MT, Birkenbach MP, Lynch M. ANCA-Associated Vasculitis Following Pfizer-BioNTech COVID-19 Vaccine. Am J Kidney Dis. 2021; 78: 611-613.

71. Sirpal V, Fichadiya H, Al-Alwan A, Ashok M. A case of fsgs in a previously healthy male after the second dose of covid-19 mrna vaccine. J. Investig. Med. 2022; 1041-1042.

72. So D, Min KW, Jung WY, Han SW, Yu MY. Microscopic polyangiitis following mRNA COVID-19 vaccination: A case report. J Korean Med Sci. 2022; 37: e154.

73. Srinivasan V, Geara AS, Han S, Hogan JJ, Coppock G. Need for symptom monitoring in IgA nephropathy patients post COVID-19 vaccination. Clin Nephrol. 2022; 97: 193-194.

74. Sugita K, Kaneko S, Hisada R, Harano M, Anno E, Hagiwara S, Imai E, Nagata M, Tsukamoto Y. Development of IgA vasculitis with severe glomerulonephritis after COVID-19 vaccination: A case report and literature review. CEN Case Rep. 2022; 11: 436-441.

75. Tan HZ, Tan RY, Choo JCJ, Lim CC, Tan CS, Loh AHL, Tien CS, Tan PH, Woo KT. Is COVID-19 vaccination unmasking glomerulonephritis? Kidney Int. 2021; 100: 469-471.

76. Thappy S, Thalappil SR, Abbarh S, Al-Mashdali A, Akhtar M, Alkadi MM. Minimal change disease following the Moderna COVID-19 vaccine: First case report. BMC Nephrol. 2021; 22: 376.

77. Timmermans SAMEG, Busch MH, Abdul-Hamid MA, Frenken LAM, Aarnoudse AJ, van Paassen P. Primary podocytopathies after covid-19 vaccination. Kidney Int Rep. 2022; 7: 892-894.

78. Unver S, Haholu A, Yildirim S. Nephrotic syndrome and acute kidney injury following CoronaVac anti-SARS-CoV-2 vaccine. Clin Kidney J. 2021; 14: 2608-2611.

79. Villa M, Díaz-Crespo F, Pérez de José A, Verdalles Ú, Verde E, Almeida Ruiz F, Acosta A, Mijaylova A, Goicoechea M. A case of ANCA-associated vasculitis after AZD1222 (Oxford-AstraZeneca) SARS-CoV-2 vaccination: casualty or causality? Kidney Int. 2021; 100: 937-938.

80. Yadav R, Shah S, Chhetri S. ANCA-associated vasculitis following Johnson and Johnson COVID-19 vaccine. Ann Med Surg (Lond). 2022; 79: 104123.

81. Yokote S, Ueda H, Shimizu A, Okabe M, Yamamoto K, Tsuboi N, Yokoo T. IgA nephropathy with glomerular capillary IgA deposition following SARS-CoV-2 mRNA vaccination: A report of three cases. CEN Case Rep. 2022; 11: 499-505.

82. Zavala-Miranda MF, González-Ibarra SG, Pérez-Arias AA, Uribe-Uribe NO, Mejia-Vilet JM. New-onset systemic lupus erythematosus beginning as class V lupus nephritis after COVID-19 vaccination. Kidney Int. 2021; 100: 1340-1341.
